# *In vivo* imaging of emerging endocrine cells reveals a requirement for PI3K-regulated motility in pancreatic islet morphogenesis

**DOI:** 10.1242/dev.158477

**Published:** 2018-02-01

**Authors:** Julia Freudenblum, José A. Iglesias, Martin Hermann, Tanja Walsen, Armin Wilfinger, Dirk Meyer, Robin A. Kimmel

**Affiliations:** 1 Institute of Molecular Biology/CMBI, University of Innsbruck, Technikerstrasse 25, A-6020 Innsbruck, Austria; 2 Johann Radon Institute for Computational and Applied Mathematics (RICAM), Austrian Academy of Sciences, Altenbergerstrasse 69, A-4040 Linz, Austria; 3 Department of Anaesthesiology and Critical Care Medicine, Innsbruck Medical University, Innrain 66, 6020 Innsbruck, Austria; 4 Department of Neurosurgery, Medical University of Innsbruck, 6020 Innsbruck Austria

**Keywords:** Filopodia, PI3K, Pancreas, Islet, Morphogenesis, Zebrafish

## Abstract

The three-dimensional architecture of the pancreatic islet is integral to beta cell function, but the process of islet formation remains poorly understood due to the difficulties of imaging internal organs with cellular resolution. Within transparent zebrafish larvae, the developing pancreas is relatively superficial and thus amenable to live imaging approaches. We performed *in vivo* time-lapse and longitudinal imaging studies to follow islet development, visualizing both naturally occurring islet cells and cells arising with an accelerated timecourse following an induction approach. These studies revealed previously unappreciated fine dynamic protrusions projecting between neighboring and distant endocrine cells. Using pharmacological compound and toxin interference approaches, and single-cell analysis of morphology and cell dynamics, we determined that endocrine cell motility is regulated by phosphoinositide 3-kinase (PI3K) and G-protein-coupled receptor (GPCR) signaling. Linking cell dynamics to islet formation, perturbation of protrusion formation disrupted endocrine cell coalescence, and correlated with decreased islet cell differentiation. These studies identified novel cell behaviors contributing to islet morphogenesis, and suggest a model in which dynamic exploratory filopodia establish cell-cell contacts that subsequently promote cell clustering.

## Abstract (precis)

**Summary:** Pancreatic endocrine cells extend previously unrecognized dynamic, flexible, fine projections to guide clustering into a compacted islet in a process regulated by PI3K and GPCR.

## INTRODUCTION

Pancreatic islets are clusters of endocrine cells that produce hormones important for regulation of glucose homeostasis. During pancreas development – a process conserved across vertebrate species – morphogenesis is coordinated with the establishment of exocrine, ductal and endocrine islet compartments. In mammalian pancreas, the ‘primary transition’ is characterized by epithelial budding and formation of early primitive endocrine cells, while the definitive endocrine cells arise during a second wave of cell expansion and differentiation called the ‘secondary transition’ (Marty-Santos and Cleaver, 2015). During the secondary transition, endocrine precursors differentiate and emerge from the pre-ductal epithelial plexus and progressively assemble into islets (Bankaitis et al., 2015; Pan and Wright, 2011).

In zebrafish, an early-forming principal islet originates from the coalescence of a population of dorsal endoderm cells, and arises prior to formation of the gut tube (Tehrani and Lin, 2011). These early endocrine cells thus form independently from pancreatic ductal tissue, and they contribute minimally to the adult endocrine mass (Hesselson et al., 2009). Secondary islet cells in zebrafish, by contrast, arise through a genetic program that is highly similar to that of secondary transition endocrine cells in mammals (Beer et al., 2016; Kimmel and Meyer, 2016). They furthermore show analogous behavior to their mammalian counterparts, as they emerge from a branching ductal network and cluster into polyclonal islets (Beer et al., 2016; Ninov et al., 2013).

Genetic studies in model organisms have successfully defined signaling pathways contributing to pancreas and islet cell type differentiation. However, owing to the experimentally inaccessible location of the pancreas in humans and most animal models, relatively little is known about the cellular dynamics and molecular mechanisms controlling islet morphogenesis. As single endocrine cells as well as clusters have been identified in pancreatic mesenchyme, islet formation has been postulated to involve cell migration in response to external directional cues (Cole et al., 2009; Puri and Hebrok, 2007; Pauerstein et al., 2017). However, the finding of islet clusters forming in close proximity to the ductal epithelium (Bader et al., 2016; Kesavan et al., 2014; Pan and Wright, 2011) suggests that cell interactions at close distances might contribute to islet formation. *In vitro*, endocrine cells have an intrinsic capacity to organize into aggregates that recapitulate many aspects of native islet structure (Woodford and Zandstra, 2012). This further implicates endocrine intercellular communication in islet formation, which could be mediated by secreted factors or cell-cell contacts.

Several pathways have been linked to islet morphogenesis based on fixed tissue and *in vitro* observations. EGFR signaling was postulated to act through Rac1 to modulate cell-cell contacts important for endocrine cell movements (Greiner et al., 2009; Miettinen et al., 2000). Furthermore, EGF is able to induce migration of pancreas-derived cells *in vitro* (Hardikar et al., 2003). Cell-cell adhesion was shown to impact islet assembly in mouse, where beta cells overexpressing a dominant-negative E-cadherin remained dispersed instead of forming clusters (Dahl et al., 1996).

A limited number of studies have used *in vivo* imaging to address mechanisms of endocrine cell clustering. Time-lapse imaging of endocrine cells in mouse pancreatic explants revealed active movements, dynamic morphologies, and aggregation of cells into clusters (Kesavan et al., 2014; Pauerstein et al., 2015; Puri and Hebrok, 2007). In addition, beta cell expression of a constitutively active Cdc42, which perturbs actin dynamics, interfered with delamination and differentiation, and reduced cell movement (Kesavan et al., 2014). Additional studies showed that blockade of G-protein-coupled receptor (GPCR) signaling resulted in a dispersed islet phenotype in mouse pancreas, and disrupted the clustering of principal islet cells in zebrafish (Serafimidis et al., 2011). In a recent report, Semaphorin signaling from the peripheral mesenchyme was suggested to promote directional movement of islet cells (Pauerstein et al., 2017).

In this work, we performed live imaging with novel transgenes to visualize endocrine cell morphologies and movements with high spatial and temporal resolution. We show that islet cells are highly motile and generate fine dynamic protrusions, and we characterize this motility at the single-cell level. In probing molecular mediators of motility and assembly, we found that disruption of protrusion formation through inhibition of PI3K is associated with perturbation of islet assembly and also blocks endocrine cell differentiation. We further demonstrate that blockade of GPCR signaling similarly inhibits cellular motility and disrupts islet formation. Our findings suggest that cell motility, regulated by PI3K and GPCR potentially acting in a common pathway, plays an important role in islet morphogenesis.

## RESULTS

### Morphology and dynamics of nascent endocrine cells

**Fig. 1.**
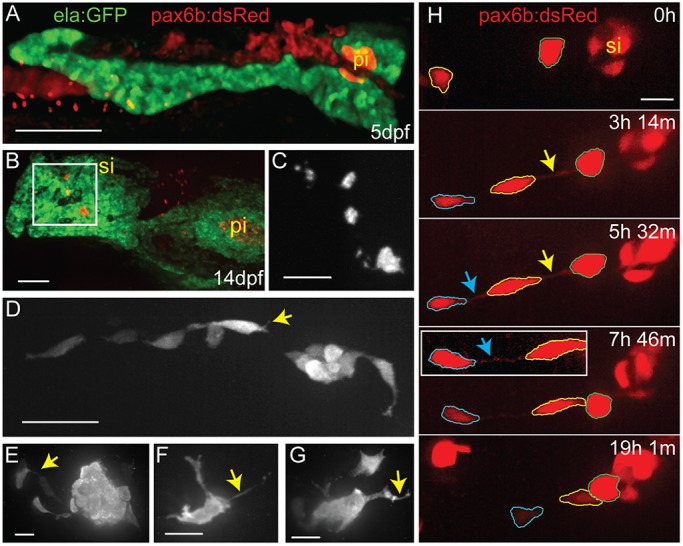
**Endocrine cells display complex morphologies during secondary islet assembly.** (A) Maximal projection of confocal stack of the pancreas at 5 dpf, imaged in a living zebrafish larva. Fluorescent transgenes label the exocrine (*ela:GFP*, green) and endocrine (*pax6b:dsRed*, red) compartments. (B) Maximal projection of confocal stack of fixed and microdissected pancreas from 14 dpf *ela:GFP;pax6b:dsRed* transgenic larva. This image was assembled by stitching together images of partially overlapping regions, using the Pairwise Stitching plug-in for ImageJ (Preibisch et al., 2009). pi, principal islet; si, secondary islet. (C) Close-up of *pax6b:dsRed*^+^ secondary islets (gray) from boxed region in B. (D-G) Confocal *z*-stack projections of secondary islet cells and clusters from samples as in B, showing *pax6b:dsRed* transgene expression (gray). Yellow arrows highlight cell protrusions. (H) Confocal image series (maximum projections) of endocrine cells in the posterior pancreas of a *pax6b:dsRed* transgenic larva beginning at 14 dpf, with subsequent images acquired at the times indicated (h, hours; m, minutes). Outlines (blue, yellow and green) indicate individual cells that move into closer proximity to each other and to a pre-existing secondary islet. Arrows indicate fine cell-cell connections. Inset, cell-cell connections become visible with contrast enhancement (blue arrow). Nonlinear gamma adjustment was applied to highlight fine protrusions and cell-cell connections. Scale bars: 100 µm in A,B; 50 µm in C; 25 µm in D; 10 µm in E-H;

During zebrafish secondary islet formation, which begins ∼5 days post fertilization (dpf), endocrine precursors differentiate from progenitors located in the intrapancreatic duct. Although it initially develops in a deep internal location, after 5 dpf the zebrafish pancreas assumes a planar form with a lateral superficial position, which is accessible to live imaging using fluorescent transgenes (Fig. 1A). As development progresses at later larval stages, the pancreas assumes a complex lobular morphology, curving around the gut (Fig. 1B).

From 6-8 dpf, secondary islet cells appear with low frequency and beta cells are rarely detected (Fig. S1A-D, Table S1) (Moro et al., 2009; Parsons et al., 2009). Cell clusters are detectable at ∼13-15 dpf and can be visualized in microdissected pancreata expressing cell type-specific transgenes (Fig. 1B,C), and in live samples by confocal microscopy. At these stages, the number of cells and islets and their distribution are highly variable between samples (Fig. S1E,F, Table S2). Secondary endocrine cells expressing transgenes driven from the pan-endocrine *pax6b* promoter (Delporte et al., 2008) are found as single cells, as well as in small and larger clusters (Fig. 1C-F, Fig. S1E,F). In fixed samples, these cells exhibit long cytoplasmic extensions, fine intercellular connections, as well as shorter filopodia (Fig. 1D-G).

The complex cell morphologies observed in fixed samples imply that cell dynamics contribute to islet morphogenesis. To explore cellular behaviors, we imaged secondary islet cells in 2-week-old (13-15 dpf) *pax6b* promoter-driven transgenics. Secondary islet cells project protrusions (Fig. S1H), which change over time (Fig. S1G,I). We observed cells tethered by narrow connections and the movement of cells into closer proximity (Fig. 1H, Fig. S1G,I,J, summarized in Table S3).

### Induced islet cells show dynamic protrusions during clustering

In naturally arising endocrine cells, the asynchronous and heterogeneous progression of islet development yields only a minority of samples at a given stage with cells in the process of clustering, and in which movements can be captured during imaging. Furthermore, optical accessibility of developing islets decreases after 8 dpf, as overlying tissues increase in thickness and opacity, and organ complexity increases. To permit studies of islet morphogenesis with conditions favorable for imaging and manipulation, precursor differentiation and secondary islet formation can be accelerated through modulation of regulatory pathways, for example by inhibition of Notch or retinoic acid signaling (Huang et al., 2014; Parsons et al., 2009; Wang et al., 2011).

We applied this approach to follow islet cell clustering by applying Notch inhibitor for 24 h starting at 4 dpf. We began imaging at 6 dpf, to provide time for cell differentiation and initiation of transgene expression, and followed samples by confocal microscopy for up to 3 days. *pax6b:dsRed* indicated the entire endocrine cell population, while beta cell morphology was highlighted by the *mnx1:memGFP* transgene, in which an *mnx1* promoter upstream of a membrane-targeted, farnesylated GFP drives expression in neurons and early beta cells (Arkhipova et al., 2012; Flanagan-Steet et al., 2005). To maintain viability over several days, samples were removed from the agarose and allowed to recover between imaging sessions. We noted progressive cell aggregation over time, with distinct clusters evident within 48-72 h (8-9 dpf). Although cluster formation was a consistent finding (*n*=9/9), cell behaviors were complex and heterogeneous, and the configuration of nascent islets varied between samples (Fig. S2).

**Fig. 2.**
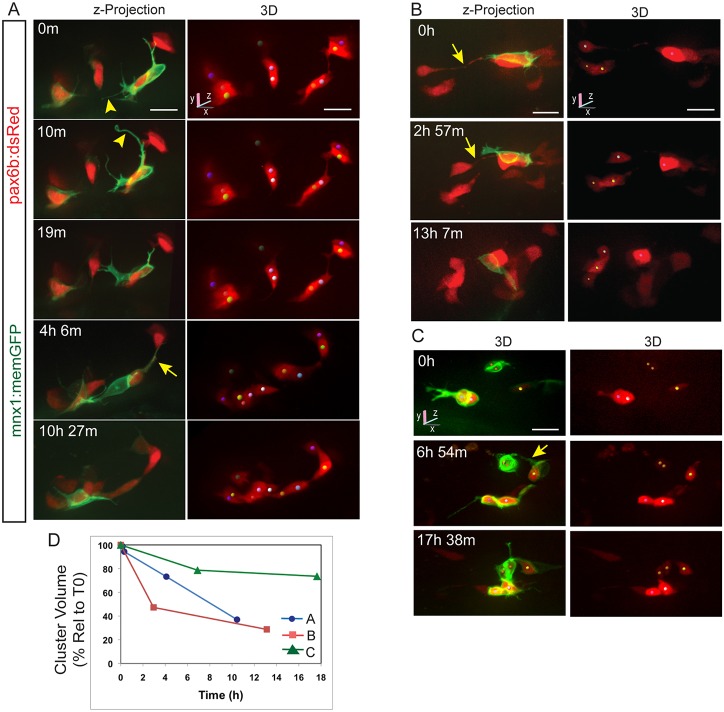
**Clustering endocrine cells extend dynamic protrusions.** (A-C) Image series acquired by confocal microscopy showing pancreatic endocrine cells in *pax6b:dsRed;mnx1:memGFP* transgenics at 7 dpf, following Notch inhibitor treatment from 4 dpf to 5 dpf. Shown are maximum intensity projections (A,B, left) and 3D representations of tracked cells (colored spheres), rotated to best visualize individual cells (A,B, right, C). *mnx1:memGFP* transgene expression delineates dynamic protrusions (arrowheads). Arrows indicate fine cell-cell connections. (D) Progression of cell clustering over time. Clustering is quantitated as the volume of a convex 3D polygon enclosing the cell centers (see Fig. S3A), plotted against time for the samples shown in A-C. Volume decreases over time as cells come into closer proximity (for details see the supplementary Materials and Methods). Scale bars: 10 μm.

**Fig. 3.**
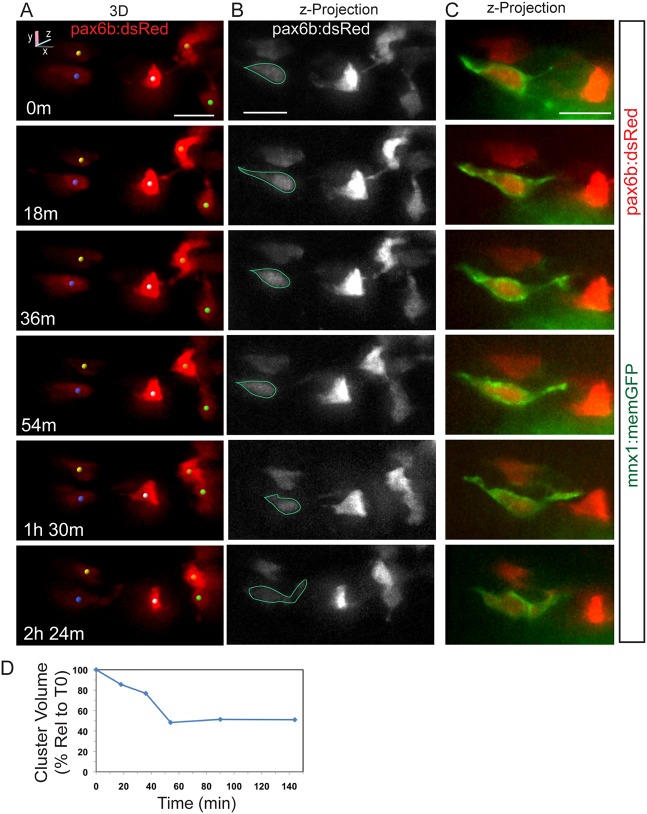
**Dynamics of cell coalescence.** (A) Confocal time-lapse series of Notch inhibitor-treated *pax6b:dsRed; mnx1:memGFP* transgenic at 7 dpf showing coalescence of two cells (orange and green spheres). Three additional cells are tracked. (B) *z*-projections corresponding to the images in A, with RFP rendered in gray. (C) Two-channel, close-up view of the GFP^+^ cell outlined in B (green). A subset of images from a series collected at 18 min intervals are shown. Scale bars: 10 µm. (D) Quantitation of clustering (as in Fig. 2D) for the tracked cells indicated in A.

To gain further insights into cellular mechanisms, we focused on cells that were loosely associated or in close proximity but not yet clustered. As in the naturally occurring endocrine cells, induced endocrine cells showed filopodia, shape changes, and narrow cell-cell connections as they moved closer together (Fig. 2A-C, Fig. 3A-C). Particularly striking was the appearance of long, flexible and highly dynamic protrusions revealed by the membrane-targeted *mnx1:memGFP* transgene, which are barely detectable by cytoplasmic dsRed expression (Fig. 2A, Fig. 3). By collecting image sequences at shorter (minutes) and longer (hours) time intervals, we captured movements of single cells and groups of cells along axes demarcated by narrow cell-cell connections (Fig. 2A-C, Fig. S3B; *n*=7/11 samples tracked over time). We further observed that as cells coalesce, narrow filopodia transform into broader connections with progressive shifting of cytoplasm (Fig. 3A,B, Fig. S4, Movie 1).

To quantitate cell clustering in complex cell configurations, we calculated the volume of a polygon enclosing the tracked cells to represent the combined distances between cells in one measure. As cells move closer together, the volume of this polygon decreases (Fig. S3A). Tracking studies showed that clustering was not uniform or constant, as examples showed variations of slope in plots of clustering versus time, both within and between samples (Fig. 2D, Fig. 3D).

### The *krt18:LifeActTom* transgene labels the actin cytoskeleton in epithelia and pancreatic duct

To further dissect the process of islet formation, we more closely examined the origin of the newly emerging cells. These cells arise from bipotent duct/endocrine progenitors, which are labeled by transgene expression driven from the Notch-responsive Tp1 element (Parsons et al., 2009; Wang et al., 2011), and are organized in a branching network that extends the length of the pancreatic tail (Fig. S5A,B).

The protrusion formation and cell shape changes observed during islet morphogenesis are indicative of dynamic rearrangements of the actin cytoskeleton. In order to better visualize actin-based cytoskeletal dynamics within the duct and emerging endocrine precursors *in vivo*, we generated transgenic fish containing the epithelial *keratin 18* (*krt18*) promoter upstream of a red fluorescent LifeAct-tandem-dimer-Tomato fusion protein *Tg(krt18:LifeActTom)*. *krt18* is expressed in intrapancreatic ducts in zebrafish (Lorent et al., 2010; Yee et al., 2005), and a previously described *krt18* promoter was shown to direct reporter gene expression to epidermis and gut (Wang et al., 2006), and the biliary system (Wilkins et al., 2014). The F-actin-binding peptide LifeAct labels F-actin filaments without disrupting endogenous actin dynamics (Phng et al., 2013; Riedl et al., 2008).

Imaging of live *krt18:LifeActTom* embryos showed labeled surface epidermis at the 15-somite stage, with enhanced signal at cell-cell boundaries (Fig. S5C,D). To localize *krt18* promoter-directed expression within the pancreas, we examined *krt18:LifeActTom* in combination with previously characterized pancreas cell type-specific transgenes. Within the exocrine pancreas region defined by expression of *Ptf1a:GFP* (Pisharath et al., 2007), a branching network of LifeActTom-expressing cells extended from the principal islet posteriorly into the pancreatic tail (Fig. S5E,F). LifeActTom expression colocalized with duct-specific *Tp1:GFP* expression (Parsons et al., 2009), and delineated the long processes that interconnect these cells (Fig. S5G,H). Expression of *krt18:LifeActTom* was also observed in blood vessels, consistent with previous reports (Schaffeld et al., 2002; Yee et al., 2005) (Fig. S5E). The *krt18:LifeActTom* transgene signal colocalized with Phalloidin staining in cell membranes of the gut epithelium (Fig. S5I,J), and with enhanced Phalloidin signal in the pancreatic duct (Navis and Bagnat, 2015) (Fig. S5K,L). *krt18:LifeActTom* further colocalized with 2F11 antibody staining, which targets the calcium-binding protein Annexin A4 (Anxa4) (Zhang et al., 2014), and in zebrafish labels the pancreatic duct and emerging endocrine cells (Matsuda et al., 2013; Zhang et al., 2014) (Fig. S5M,N).

### Emerging endocrine cells project fine filopodia

**Fig. 4.**
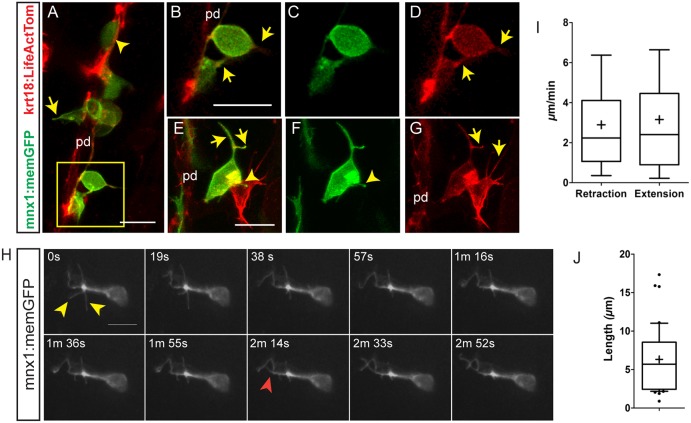
**Emerging endocrine cells extend protrusions.** Confocal image stacks of differentiating pancreatic endocrine cells in living 6 dpf *krt18:LifeActTom;mnx1:memGFP* transgenics following Notch inhibitor treatment from 4 dpf to 5 dpf. Shown are maximum intensity projections (A,E-G) and single *z*-slices (B-D). pd, pancreatic duct. (A) A rounded GFP^+^/LifeActTom^+^ early beta cell found within the duct (arrowhead); the cell leaving the duct has a narrow protrusion (arrow). (B-D) Close-up of the boxed region from A, showing merged (B) and single channels (C,D). GFP^+^/LifeActTom^+^ early beta cells extend actin-rich processes (arrows). (E-G) Thin actin-rich processes (arrows) project and connect two LifeActTom^+^ cells (arrowhead) in close proximity to the duct. The GFP^+^ cell is a nascent beta cell, whereas the GFP^−^ cell is likely to represent a differentiating non-beta endocrine cell. (H) Maximum intensity projections at the indicated time points of time-lapse series acquired by spinning disc microscopy of Notch inhibitor-treated larva analyzed at 6 dpf. Nonlinear gamma adjustment was applied to enhance low contrast signals. Dynamic extension (red arrowhead) and retraction (yellow arrowheads) of fine protrusions were detected. (I) Quantitation of instantaneous retraction and extension rates analyzed from images captured as in H. (J) Maximal filopodia lengths from image series as in H. In box-whisker plots, whiskers represent 10-90%, outliers are indicated by dots, and the mean is indicated by ‘+’. Sample details are provided in the supplementary Materials and Methods. Scale bars: 10 µm in A-H.

Having established that the *krt18:LifeActTom* transgene indicated actin distribution in the pancreatic duct of living embryos, we next examined nascent beta cells emerging from, and in proximity to, the pancreatic duct. In the pancreas of Notch inhibitor-treated *krt18:LifeActTom;mnx1:memGFP* transgenic larvae at 6 dpf, we could distinguish LifeActTom^+^/memGFP^+^ early beta cells still within the duct that were relatively round and showed diffuse signal from both GFP and LifeActTom (Fig. 4A). Beta cells emerging from the duct extended fine actin-rich protrusions directed away from the duct as well as along the duct, connecting to nearby emerging cells (Fig. 4A-D). An enrichment of actin signal was detected at the point where the cell remains attached to the duct (Fig. 4B-D). LifeActTom^+^/memGFP^+^ cells that were detached from the duct also projected actin-rich protrusions, which made contact with nearby cells (Fig. 4E-G). These studies implicate actin-based cytoskeletal rearrangements not only in delamination, consistent with previous studies (Kesavan et al., 2014), but also in performing important functions in cells that have left the duct and are beginning to cluster.

As fine protrusions can serve various functions depending on their morphological properties (Jacquemet et al., 2015), we analyzed filopodial dynamics in single cells. *mnx1:memGFP*-positive early beta cells, located in proximity to the duct defined by *krt18:LifeActTom* expression, were examined with spinning disc confocal microscopy, with image *z*-stack capture at time intervals ranging from 12 to 20 s. These studies revealed highly dynamic, small diameter (<2 µm) protrusions, that displayed undulations and branching (Fig. 4H, Movie 2). The majority (75%) had a maximal extension of less than 9 µm, but filopodia in some cases (3/41 examined) reached lengths greater than 15 µm (Fig. 4J). Mean velocities of extension (3.2 µm/min) and retraction (2.9 µm/min) were similar (unpaired *t*-test, *P*=0.3), with maximum speeds of greater than 6 µm/min (Fig. 4I). Filopodial length and extension/retraction velocities resemble those seen *in vivo* in endothelial cells (Yu et al., 2015) and *in vitro* in neuronal growth cones (Mallavarapu and Mitchison, 1999). Overall, these analyses revealed dynamic, flexible protrusions produced by clustering endocrine cells, which show characteristics of exploratory filopodia with function in environmental sensing and cell-cell recognition.

To demonstrate that similar protrusions are found in secondary endocrine cells arising during the normal course of development, we first imaged *mnx1:memGFP*-expressing cells in uninduced samples at 8 dpf, which are only rarely detectable (*n*=7/45 larvae). In 4/7 samples, the signal was too weak to distinguish cell morphology. In 2/3 samples containing cells with sufficiently strong signal, fine dynamic protrusions were detected (Fig. S6A-D). At 2 weeks (13-15 dpf), more cells show *m*n*x1:memGFP* expression (Table S1), and dynamic protrusions could be observed from cells in isolation and already in clusters (Fig. S6E-G; 6/14 samples imaged).

The above studies highlighted protrusions in *mnx1:memGFP*-positive beta cells and *pax6b* transgene-expressing endocrine cells. To show that dynamic morphologies are also characteristic of non-beta endocrine cells, we performed time-lapse imaging of *glucacon a* (*gcga*)-expressing alpha cells using *gcga:GFP* transgenics (Zecchin et al., 2007) at 7 dpf following endocrine cell induction. *gcga:GFP*^+^ cells displayed protrusions and active changes in cell shape (*n*=4/4 time-lapse movies, Fig. S6H).

### PI3K regulates protrusion formation in aggregating endocrine cells

**Fig. 5.**
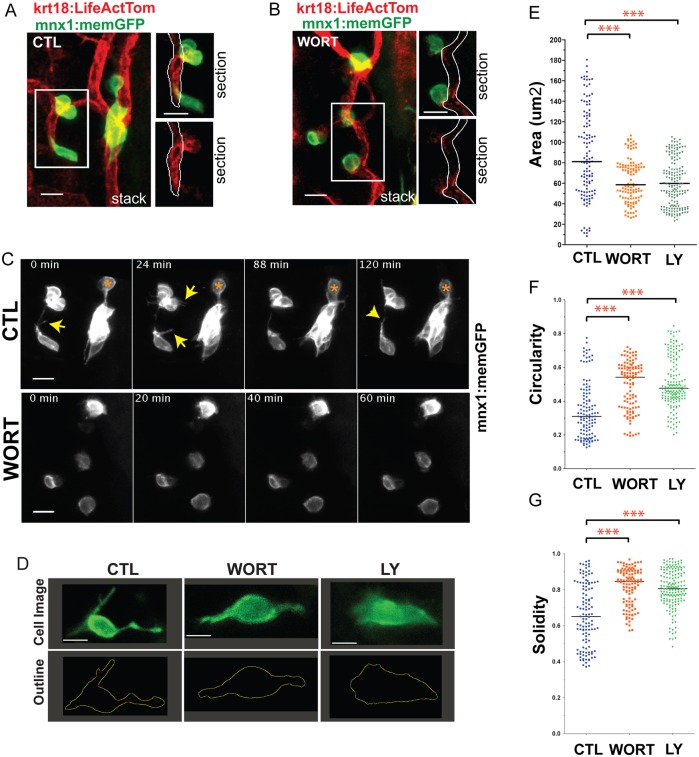
**PI3K inhibition impacts cell morphologies and dynamics.** (A,B) (Left) Maximum intensity projections of confocal image stacks of early beta cells in (A) control (CTL) and (B) Wortmannin-treated (WORT) 7 dpf *krt18:LifeActTom;mnx1:memGFP* transgenics following islet induction at 4 dpf. (Right) Single *z*-planes from the boxed regions of the image stack (top, merge; bottom, single channel, LifeActTom) with the duct indicated (white outline). (C) Selected time points from time-lapse image series of samples from A and B. Arrows indicate protrusions. Asterisk indicates a cell that moves closer to the cluster. (D) Single images from time-lapse series, showing individual nascent beta cells in *mnx1:memGFP* transgenics treated to induce secondary islets from 4-5 dpf, followed by treatment at 6 dpf as indicated (WORT, 100 nM Wortmannin; LY, 50 µm Ly294002; CTL, DMSO), for 3-4 h prior to imaging (top row). The lower panels show the cell outline used for quantitative morphology analysis. (E-G) Cell area (E), circularity (F) and solidity (G) measured in individual frames of time-lapse series (see Fig. S3). ***P<0.0001, one-way ANOVA with Dunnett's post-test. CTL, 14 embryos, 118 time points; WORT, 14 embryos, 111 time points; LY, 13 embryos, 162 time points. Additional sample information is provided in Table S4. Scale bars: 10 µm in A-C; 5 µm in D.

Phosphoinositide 3-kinase (PI3K) has a crucial and highly conserved role in cell motility in many cell types, controlling the location of actin polymerization and thereby the orientation and shape of protrusions (Leslie et al., 2008). To examine the contribution of PI3K to islet cell dynamics, *mnx1:memGFP;krt18:LifeActTom* larvae were first treated from 4-5 dpf with Notch inhibitor, then at 7 dpf with the irreversible PI3K inhibitor Wortmannin (WORT) and observed by time-lapse microscopy for cell morphology and protrusion dynamics. Control *mnx1:memGFP*^+^ cells in the vicinity of the pancreatic duct (Fig. 5A) extended and retracted processes both towards and away from neighboring cells (Fig. 5C, top; Movie 3). By contrast, in samples treated with WORT for 3-4 h prior to imaging, GFP^+^ cells produced fewer protrusions and there were few contacts between neighboring cells (Fig. 5B,C, bottom; Movie 4).

To more precisely define the effect of PI3K inhibition on cellular morphology and dynamics, we applied quantitative measures of cell shape to sequential frames within time-lapse image series captured at 6 dpf, focusing on isolated single cells (Fig. 5D, Fig. S7A). For these studies, we applied WORT and, in addition, used the reversible PI3K inhibitor Ly294002 (LY). We used the parameter solidity as a quantitative indicator of the extent of filopodia formation (Xue et al., 2014), as it is amenable to efficient and unbiased image analysis approaches (Barry et al., 2015). Solidity, which is the ratio of cell area to convex hull area, has a maximum of one for a regular round object and this value decreases towards 0 in proportion to protrusion formation (Fig. S8A). In single cells traced over time, cell morphologies, as revealed by cell solidity, circularity and area, differed between control and treatment groups (Fig. S8B-D, Table S4). The high area, low solidity phenotype of individual cells in control samples was maintained over the time of imaging, suggesting that sustained periods of active motile behaviors are a feature of islet cell coalescence. Since individual cells were followed for variable times due to technical issues of tissue movement and signal bleaching, we combined all time points from all cells for statistical analysis (Fig. 5E-G). Overall, following PI3K inhibition, beta cells in the pancreatic tail were more compact, with decreased cell area and higher circularity compared with controls (Fig. 5E,F). Consistent with our observation of decreased protrusion formation following PI3K inhibition, solidity was significantly higher in treated cells than in controls (Fig. 5G).

To assess the effects of PI3K inhibition on overall membrane motility, we examined the expansion and retraction of cell membranes using an analysis approach that defines the cell body excluding fine protrusions (Tsygankov et al., 2014) (Fig. S7B). When overall membrane motility was considered, there was no reduction in the PI3K inhibitor-treated groups as compared with controls (Fig. S7C). These studies showed that global membrane motility was maintained when PI3K was inhibited, and suggest that PI3K preferentially regulates fine protrusions.

### Quantitative assessment of islet cell clustering

Our studies above indicate that islet formation, both naturally occurring and when accelerated by Notch inhibition, occurs in an asynchronous, non-stereotypical fashion. To assist in identifying molecular pathways acting in islet formation, it was necessary to make global assessments and quantify perturbations. Owing to the comparatively small size of zebrafish and the accessible location of the pancreas during early larval stages, one can readily image the entire pancreas in the living animal (Fig. 1A). Efficient and consistent processing of many samples is facilitated by capturing the entire pancreas in one image, which enables imaging sufficient numbers of samples (at the same stage) for meaningful quantitative comparisons.

To establish a quantitative method to assess islet formation, we induced islets at 4 dpf and then imaged the whole pancreas at 6 dpf, by which time endocrine cells can be detected, and also at 8 dpf, when clusters have formed (Fig. S9A). Emerging secondary islet cells were detected through nuclear *Tp1:H2BmCherry* transgene expression (Ninov et al., 2012), combined with *pax6b:GFP* to indicate newly generated endocrine cells (Delporte et al., 2008). At 6 dpf, newly differentiated GFP^+^/H2BmCherry^+^ double-positive cells were distributed predominantly as single cells (Fig. S9B-E, *n*=7 larvae). Forty-eight hours later, at 8 dpf, the GFP^+^ cells retained the long-lived H2BmCherry in their nuclei but were arranged in larger clusters (Fig. S9F-I, *n*=7 larvae), consistent with our previous observations (Fig. S2). Quantitation of secondary islet three-dimensional volumes showed a significant increase in the size of islets between 6 dpf and 8 dpf (Fig. S9E,I,J). Islet volumes at 8 dpf thus represent a robust quantitative phenotype, despite the overall heterogeneity of islet morphogenesis.

We then examined whether cell division contributes to the increase in cell cluster size, by comparing endocrine cell number in the pancreatic tail at 6 dpf and 8 dpf. Using the H2BmCherry nuclear label to facilitate counting of differentiated endocrine cells, we determined that the number of GFP^+^/H2BmCherry^+^ secondary islet cells did not change significantly between 6 dpf and 8 dpf (Fig. S9K). This is consistent with previous findings that differentiation towards an endocrine fate is associated with cell cycle exit (Bechard et al., 2016; Ninov et al., 2012). This implied that cell proliferation contributed minimally, if at all, to the increase in cluster size.

### PI3K impacts islet cell coalescence

**Fig. 6.**
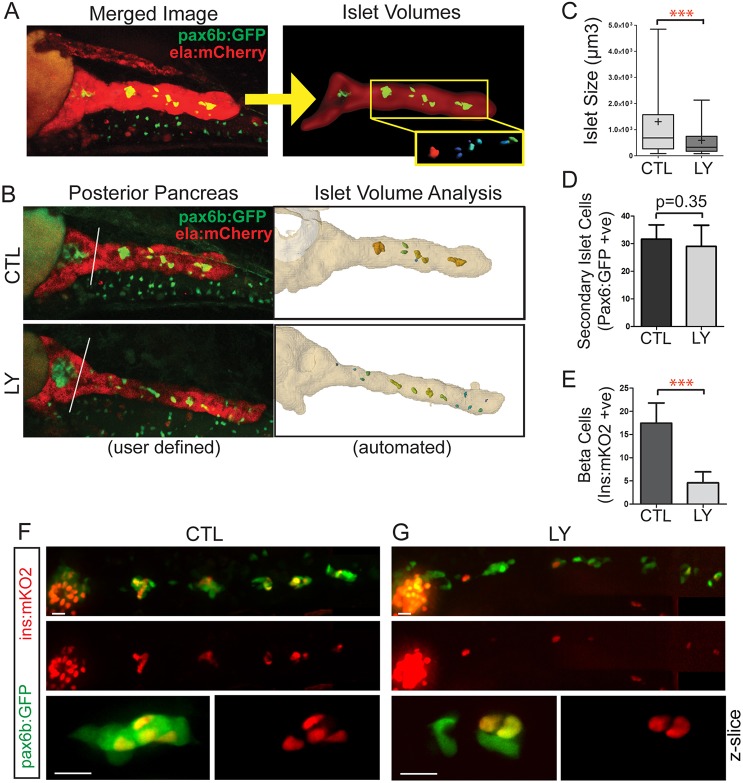
**PI3K inhibition interferes with islet assembly assay.** (A) Secondary islet analysis is based on the identification and three-dimensional segmentation of exocrine pancreas as labeled by *ela:mCherry* expression. (B) With our automated script, the user defines the posterior pancreas based on a composite image of the middle slice of *ela:mCherry* (red channel) and a *z*-projection of the *pax6b:GFP* image stack (left, white line). The custom software automatically delineates the whole pancreas and secondary islets (right). (C) Quantitation of secondary islet size of the samples analyzed in Fig. S10B-D, using the automated method. In box-whisker plots, box extends from the 25th to 75th percentile, whiskers indicate 5th and 95th percentile, line at median, ‘+’ indicates mean. ***P<0.001, Mann–Whitney test (one-tailed). CTL, *n*=10 larvae, 133 objects, LY, *n*=8 larvae, 80 objects. (D) *pax6b:GFP*^+^ cells in the posterior pancreas of embryos treated as in C. Mean±s.d. CTL, *n*=13; LY, *n*=9. Unpaired *t*-test. (E) *ins:mKO2*^+^ cells at 8 dpf in samples treated as in C. CTL, *n*=16; LY, *n*=14. Mean±s.d. Unpaired *t*-test, ***P<0.0001. (F,G) Representative images of 8 dpf *pax6b:GFP;ins:mKO2* larvae. Control (F) or treated from 6 dpf to 8 dpf with 15 μM Ly294002 (G). Overview images (F,G, top and middle) were assembled by stitching together images of partially overlapping regions using the Pairwise Stitching plug-in for ImageJ (Preibisch et al., 2009). Scale bars: 50 µm. (F,G, bottom) Single slices from *z*-stacks of higher magnification images. Scale bars: 10 µm.

Having validated a quantitative assay for examining islet cell clustering, we used this approach to provide evidence for the hypothesis that PI3K-regulated cell motility is required for islet cell aggregation. We applied a combination of Notch inhibitor Ly411575 and retinoic acid inhibitor DEAB to robustly induce a population of secondary islet cells at 4 dpf (Huang et al., 2014), with minimal toxicity. In addition to performing manual image analysis, we optimized imaging parameters and developed an analysis pipeline for automated detection of secondary islets (Fig. 6A,B, Fig. S10A). In *pax6b:GFP;ela:mCherry* double transgenics, *ela:mCherry* delineated the exocrine pancreas, thus defining the region that contained secondary islets. For validation of our automated analysis in comparison with manual measurement, we applied the small molecule PI3K inhibitor LY. Consistent with PI3K-regulated motility having a role in islet cell coalescence, treatment of *pax6b:GFP;ela:mCherry* double transgenics with LY in our assembly assay significantly decreased islet volumes as determined by manual ImageJ analysis (Fig. S10B-D), and closely matching results were obtained using the automated algorithm (Fig. 6C).

### PI3K inhibition impacts differentiation but not cell number

PI3K is a central regulator of cell survival and proliferation, in addition to its roles in cell motility (Leslie et al., 2008). Therefore, it was necessary to confirm that equivalent numbers of secondary islet cells were maintained under control and LY treatment conditions, so as to rule out confounding influences from cell proliferation or cell death. We counted individual GFP^+^ endocrine cells in the pancreatic tail in control and LY-treated larvae at 8 dpf, following islet induction treatment as performed for islet volume analyses. We found no significant difference in GFP^+^ cells within the pancreatic tail at 8 dpf (average of 32±5 cells in control samples, *n*=13; 29±8 cells in LY-treated samples, *n*=9; *P*=0.35, unpaired *t*-test; Fig. 6D). This supports our hypothesis that PI3K is influencing islet morphogenesis and not impacting cell number.

At 6 dpf following endocrine cell induction, endocrine hormones insulin and glucagon are barely detectable (Fig. S11A,C,D), but expression is increased at 7 dpf in nascent clusters (Fig. S11B,E), thus showing a temporal association between the progression of clustering and cell differentiation in our system. We then determined whether PI3K inhibition, with its effect on islet assembly, also had an effect on cell differentiation. To count beta cells, we used a transgenic line based on the rapidly maturing, nuclear-localized mKO2-zCdt1 fusion protein (Sugiyama et al., 2009), expressed under control of a 1.2 kb *insulin* (*ins*) promoter (Huang et al., 2001; Moro et al., 2009). *ins:mKO2* transgenic larvae were treated to induce islets, followed by addition of LY or DMSO alone as a control. Supporting the hypothesis that islet assembly promotes beta cell differentiation, the number of *i*n*s:mKO2*^+^ beta cells in LY-treated larvae was significantly reduced compared with controls at 8 dpf (17±4 cells in controls, *n*=16; 5±2 cells in LY-treated samples, *n*=14; *P*<0.0001, unpaired *t*-test; Fig. 6E-G). To determine whether other endocrine cell types were affected, we performed antibody staining for Ins in combination with antibodies against glucagon (Gcg) and somatostatin (Sst). The combined number of Gcg^+^ and Sst^+^ cells was reduced to a similar degree to that of Ins^+^ cells (Fig. S11F-J). This supports a disruption of differentiation, rather than a fate change of Ins-expressing cells into a different endocrine cell type.

### The ductal progenitor plexus is re-established following islet induction

While PI3K inhibition changed the morphology of endocrine cells, the effect of PI3K inhibition on adjacent structures might also impact islet cell clustering. To avoid potential confounding effects of islet induction on duct morphology, we examined the effect of PI3K inhibitor treatment alone. *krt18:LifeActTom;Tp1:GFP* double transgenics were treated from 6 dpf to 7 dpf with LY. Imaging of native fluorescence in living larvae did not show any alteration in duct morphology (Fig. S12).

We then examined how islet induction affects the Notch-responsive progenitor cell network. Following endocrine cell induction, as many duct-associated progenitor cells convert to endocrine cells and begin to cluster, ductal structure appears disorganized, with decreased branching (Fig. S13A,B). To observe the later consequences, following treatment at 4 dpf, islet-induced transgenic larvae were grown in parallel with control samples. At 14 dpf, larvae were fixed and immunostained for pancreatic lineage transgenic markers. In *ela:GFP;pax6b:dsRed* transgenics, exocrine pancreas appeared similar between control and induced samples (Fig. S13C,D). Secondary islets could be detected in control samples based on *pax6b:dsRed* expression, whereas treated samples, as expected, showed increased secondary islets (*n*=3, Fig. S13C,D). In 14 dpf *Tp1:GFP;ela:mCherry* transgenics that had been treated for endocrine cell induction at 4 dpf, a ductal network, similar to that of untreated controls, was detected (*n*=8, Fig. S13E,F). Thus, although endocrine cell induction alters the architecture of the progenitor plexus, it appears to be restored during subsequent development.

### EGFR blockade and Rac1 inhibition do not affect islet assembly

PI3K can be activated by tyrosine kinase receptors, and has downstream effects on cytoskeleton-regulating Rho family GTPases (Artemenko et al., 2014; Shewan et al., 2011). EGF signaling, potentially acting through Rac1, was suggested to regulate islet morphogenesis (Greiner et al., 2009; Miettinen et al., 2000). To examine whether these factors act in a pathway with PI3K to influence islet cell aggregation, we utilized Tyrphostin AG 1478 and NSC 23766 to inhibit EGFR and Rac1, respectively (Gao et al., 2004; Goishi et al., 2003). Following islet induction from 4-5 dpf, inhibitors were applied for 48 h (from 6 dpf to 8 dpf), and our automated analysis method was applied. We used a well-tolerated lower dose (100% survival), and a higher dose where toxicity was seen at the end of 48 h (Table S7). EGFR or Rac1 inhibition did not impact islet size at either dose tested (Fig. S14A,B), suggesting that EGFR signaling and Rac1 activity do not influence islet cell clustering. As we focused on later islet assembly events, we do not exclude a role for these proteins in islet cell delamination in our system. We also cannot rule out compensation for Rac1 inhibition by other RhoGTPase family members.

### GPCR signaling blockade perturbs islet formation

**Fig. 7.**
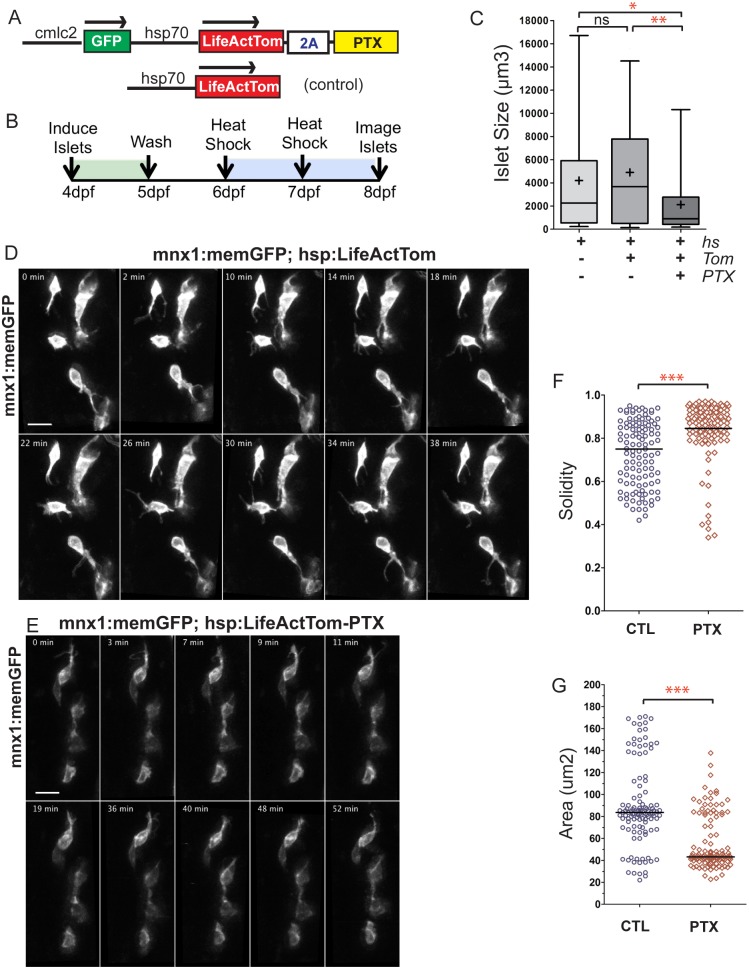
**GPCR inhibition disrupts islet assembly.** (A) Transgene *hsp70:LifeActTom-PTX* was used to inducibly express pertussis toxin (PTX, top). The transgene *hsp70:LifeActTom* served as a control (bottom). (B) Scheme of islet assembly experiment with heat shock induction of PTX. (C) Islet volume quantitation of heat shock-treated controls (*n*=12, 99 objects), compared with larvae with induced expression of LifeActTom (*n*=14, 120 objects), or LifeActTom-PTX (*n*=6, 61 objects). Images were captured on a Zeiss LSM5 and volume quantitation performed using ImageJ (minimum object size 100 µm^3^). *P<0.05, **P<0.01, Kruskal–Wallis test followed by Dunn's multiple comparison test. ns, not significant. Results are representative of two independent experiments. (D,E) Selected confocal projections from time-lapse series of control (D) and PTX-expressing (E) transgenic larvae at 7 dpf, 4 or more hours following a heat shock. Scale bars: 10 μm. (F,G) Quantitation of cell solidity (F) and area (G) measured in individual frames of time-lapse series with images acquired at 4 min intervals (as shown in Fig. S16A,B). ***P<0.0001, Mann–Whitney test, two-tailed. Data are combined from a total of eight control and eight PTX-expressing larvae analyzed in three separate experiments. Control samples include non-heat-shocked *hsp70:PTX* transgenics, and LifeActTom^+^ heat-shocked samples. Additional sample information is provided in Table S5.

PI3K can also be activated by GPCR signaling (Artemenko et al., 2014), and previous studies implicated GPCR signaling in islet morphogenesis using mouse explants and transient misexpression in early zebrafish principal islet beta cells (Serafimidis et al., 2011). To test whether blockade of GPCR signaling results in a secondary islet assembly phenotype similar to PI3K inhibition, we generated transgenic fish expressing the G protein-inhibiting pertussis toxin (PTX) under the control of a heat shock-inducible promoter, in a vector that co-expresses *LifeActTom*, and a heart-specific *cmlc2:GFP* cassette as an indicator of transgenesis (Fig. 7A, top). Functionality of this construct was confirmed by performing a heat shock at 50% epiboly and examining transgenic embryos at 24 h post fertilization (hpf). Robust ubiquitous LifeActTom expression was associated with dramatically reduced expression of cardiac *cmlc2:GFP*, and with shortening and curvature of the body axis (Fig. S15A), consistent with the previously described role of GPCR signaling in early embryogenesis and cardiac development (Pauli et al., 2014; Scott et al., 2007).

To examine the effects of GPCR blockade on islet formation, we induced secondary islets in *hsp70:LifeActTom-PTX;pax6b:GFP* double-transgenic larvae at 4 dpf, and then applied a heat shock at 6 dpf and 7 dpf (Fig. 7B). Heat-shocked samples containing an *hsp70:LifeActTom* transgene or *pax6b:GFP* alone served as controls. Quantitation of islet volumes at 8 dpf showed that cell clustering was significantly reduced in PTX-expressing embryos as compared with controls (Fig. 7C, Fig. S15B).

Given the similar impact of PI3K inhibition and GPCR blockade on islet cell aggregation, we examined whether protrusion formation and cell motility were affected in endocrine cells of PTX-expressing larvae. *hsp70:LifeActTom-PTX;mnx1:memEGFP* embryos induced by Notch inhibition were heat shocked at 7 dpf, then examined 4-6 h later by time-lapse microscopy. Similar to our findings following application of PI3K inhibitors, PTX-expressing cells appeared rounder with reduced protrusion formation, whereas control cells showed active extension and retraction of fine protrusions (Fig. 7D,E, Fig. S16A,B, Movies 5 and 6). Quantitation of morphology parameters in time-lapse image series of single cells (Fig. S16A,B) corroborated that PTX-expressing cells had higher solidity and significantly reduced area (Fig. 7F,G, Fig. S16C,D).

To examine the impact of PTX expression on endocrine cell differentiation, we determined insulin-expressing and glucagon-expressing cell number using *ins:GFP* (Pisharath et al., 2007) and *gcga:GFP* transgenics. Transgene-expressing cells in the pancreatic tail were counted at 8 dpf, following islet cell induction and heat shock treatments as performed for assessing islet assembly (Fig. 7B). There was a minor, but significant, reduction in both insulin-expressing and glucagon-expressing cells upon PTX expression (Fig. S16E-H).

## DISCUSSION

In this work, we show that endocrine cells form highly dynamic fine protrusions, and we provide evidence that this protrusion formation is important for islet assembly. Moreover, we define the morphological properties of these protrusions, establishing analogy to other tissues in which filopodia act as exploratory structures to facilitate cell-cell recognition and initiate tissue coalescence. Furthermore, the above studies define roles for GPCR signaling and PI3K in regulating the endocrine cell dynamics that occur during islet assembly.

Previous studies (Moro et al., 2009; Parsons et al., 2009) and this current work document the slow accumulation of new islet cells beginning at ∼5-6 dpf, with cell clusters being detectable by 13-14 dpf. To develop a system in which islet morphogenesis can be readily and consistently detected within a relatively short time frame, we applied an induction treatment based on endogenous signaling pathways that act during development. In this way, clustering endocrine cells can be visualized with high resolution in sufficient numbers for quantitative analyses, and the animals are young enough to take up compounds supplied in the incubation medium to probe modulators of islet clustering.

Several lines of evidence support our hypothesis that processes detected in induced islets are relevant for normal islet clustering. Examples of endocrine cell protrusions in fixed samples, and of cell movements and coalescence in live imaging, have been reported in uninduced zebrafish larvae (Kimmel et al., 2011; Ninov et al., 2012, 2013). In the current work, we built on these prior studies to present a detailed analysis of cell dynamics during islet morphogenesis within living animals. In mice, the deep internal location of the pancreas precludes *in vivo* imaging, so explant culture systems are used. Similar to our observations in zebrafish, clustering endocrine cells in explants make protrusions and show dynamic morphologies coincident with cluster formation (Bechard et al., 2016; Pauerstein et al., 2015; Puri and Hebrok, 2007; Kesavan et al., 2014). The considerable number of cells induced to cluster in our model creates a situation similar to that in mouse, where large numbers of cells differentiate during the secondary transition (Marty-Santos and Cleaver, 2015). It was difficult, although possible, to visualize filopodial dynamics in naturally arising endocrine cells in zebrafish, as only rare samples showed sufficiently strong fluorescent signal. Overall, cell behaviors during islet morphogenesis are similar in naturally occurring and induced cells in zebrafish, and furthermore appear to be conserved between zebrafish and mammals.

In many systems, filopodia play an important role as exploratory sensors in mediating cell-cell recognition and initiating cell-cell adhesion (Mattila and Lappalainen, 2008). This has been described in *Drosophila* dorsal closure (Jacinto et al., 2000), zebrafish blood vessel anastomosis (Phng et al., 2013), mammalian blastula compaction (Fierro-González et al., 2013), and recently in mouse neural tube closure (Rolo et al., 2016). In both *Drosophila* and vertebrate models, it has been proposed that filopodial contacts can transmit a mechanical pulling force to draw cells together (Fierro-González et al., 2013; Jacinto et al., 2000; Millard and Martin, 2008). The endocrine cell filopodia we describe show similarities to those of neuronal growth cones and vascular endothelial cells (Mallavarapu and Mitchison, 1999; Yu et al., 2015). We propose that endocrine islet cell filopodia scan the environment to identify and establish contacts with neighboring islet cells and initiate coalescence.

In the above studies, we focused on early events following simultaneous induction of a pool of differentiated cells, which resembles the robust endocrine differentiation during the secondary transition in mouse (Bankaitis et al., 2015). It remains possible that single-cell directional migration guides new endocrine cells to established islets later during postnatal growth and in adulthood, when the rate of new cell emergence is dramatically reduced (Ackermann and Gannon, 2007). Movement of islets away from the duct may occur through a combination of exocrine tissue expansion and active directional migration (Pauerstein et al., 2017).

GPCRs are key signal transducers impacting cell morphology and motility (Cotton and Claing, 2009). Previous studies found that GPCR signaling plays a role in islet morphogenesis using mouse *in vivo* and explant models, as well as by examining principal islet formation in zebrafish (Serafimidis et al., 2011). Consistent with this earlier work, we found that disruption of GPCR signaling by expression of PTX disrupted clustering of secondary islet cells, which are analogous to mammalian secondary transition cells. We further demonstrate changes in cell motility resulting from inhibition of GPCR signaling, providing a potential mechanistic link between this signaling pathway and morphogenetic consequences. Serafimidis et al. (2011) found no change in endocrine cell specification following PTX treatment, based on c-peptide and glucagon expression, whereas we detected a minor decrease in insulin- and glucagon-expressing cells. This discrepancy could be due to technical differences in the experimental approaches. Taken together, these results suggest that PI3K activity and GPCR signaling in endocrine cells affect differentiation to varying degrees, conceivably through discrete mechanisms.

Upon activation, GPCRs signal through heterotrimeric G-proteins, whereby both Gα and Gβγ components interact with downstream signaling pathways (Cotton and Claing, 2009; Syrovatkina et al., 2016). Membrane-associated PI3K can be activated by GPCR signaling (Cotton and Claing, 2009; Zhao and Vogt, 2008), locally generating phosphoinositides (PIs), which in turn modulate the actin cytoskeleton in discrete subcellular domains (Shewan et al., 2011). Signaling lipids have been specifically implicated for directing filopodia that scan the environment and initiate cell-cell contact, for example in wound healing (Li et al., 2013; Pickering et al., 2013). In future studies it will be important to define the relative roles of Gα and Gβγ in islet morphogenesis, and to identify additional signals that coordinate cytoskeletal dynamics, and mediate cell-cell recognition and clustering. Our studies do not exclude the possibility that differentiation impacts clustering, or that insulin itself serves as a signal contributing to islet assembly.

Screening of chemically active compounds for their biological effects in zebrafish embryos and larvae is a rapidly expanding field, which has been recently applied to identify modulators of beta cell differentiation (Rovira et al., 2011; Wang et al., 2015). Here, we described an assay for islet assembly with an automated image analysis pipeline that can be applied for compound screening, as it involves minimal manipulation, with transgenes conveniently providing a phenotypic readout in the living animal.

The generation of replacement islets from stem or other progenitor cells is a major goal for the realization of regenerative therapies for diabetes. Cell-cell interactions that occur during formation of the three-dimensional islet are crucial for beta cell function *in vitro* or *in vivo.* In this study, live imaging approaches revealed previously unappreciated protrusion dynamics of endocrine cells during islet coalescence. Through pharmacological approaches combined with a novel quantitative assay, we established that PI3K directs actin-based cell motility, and acts along with GPCR signaling to regulate islet morphogenesis. Improving our understanding of islet morphogenesis can be applied to optimizing the production of functional beta cells from undifferentiated progenitors *in vitro* and for directing *in vivo* differentiated endocrine cells to form islets.

## MATERIALS AND METHODS

### Zebrafish transgenic lines and maintenance

Zebrafish (*Danio rerio*) were maintained using standard protocols. Embryos were grown in 0.0015% PTU (Sigma, P7629) to reduce pigmentation. Larvae to be studied at 2 weeks were kept in Petri dishes until 5 dpf, then transferred to our fish facility until the time of harvest. A list of transgenic lines used in this study is provided in Table S6. Component elements were assembled using the Gateway system (Kwan et al., 2007) and transgenic lines were generated using standard techniques (for details, see the supplementary Materials and Methods).

This study was approved by the Austrian Bundesministerium für Wissenchaft und Forschung (GZ BMWFW-66.008/0007-II/3b/2012, GZ BMWFW-66.008/0009-WF/II/3b/2014, and GZ BMWFW-66.008/0018-WF/V/3b/2017). All procedures were carried out in accordance with approved guidelines.

### Compound treatments and heat shock

For time-lapse analyses, secondary islets were induced using 10 µM Ly411575 from 4-5 dpf. For cell morphology studies, larvae were treated for 3-4 h with 100 nM Wortmannin or 50 µM Ly294002. For the islet assembly assay, embryos were treated with 10 µM Ly411575 plus 25 µM DEAB (Huang et al., 2014). Embryos were treated in E3 medium (5 mM NaCl, 0.17 mM KCl, 0.33 mM MgSO_4_, 0.33 mM CaCl_2_, pH 7.5, in ddH_2_O) supplemented with 1% DMSO and 1× antibiotic/antimycotic solution (Sigma). The following compounds were used: Ly411575 (Sigma, SML0506), Wortmannin (Sigma, W1628), Ly294002 (Antibodies-Online, ABIN412265), Tyrphostin AG 1478 (Sigma, T4182) and NCS23766 (Tocris, 2161). Stock solutions were prepared in DMSO, except for NCS23766 in ddH_2_O. Wortmannin was stored aliquotted at −80°C; for the remaining compounds, aliquots were stored at −20°C. For experimental doses, we were guided by published studies (Sabha et al., 2016; Yoo et al., 2010) and additional testing in our system. Dilution studies were performed by treating pools of 10-15 embryos in E3 medium containing varying compound concentrations, to determine an optimal dose for 48 h of treatment (Table S7, Fig. S17). Samples were observed for lethality, or changes in morphology or movement. Embryos carrying *hsp70:LifeActTom-PTX* or *hsp70:LifeActTom* were heat shocked at 6 dpf and 7 dpf for 20 min at 38°C in a shaking waterbath.

### Antibody staining

Larvae were harvested at 7 dpf, fixed for 1 to 2 h at room temperature in 4% paraformaldehyde (PFA)/1% DMSO in PBS, then washed three times for 5 min each with 1× PBS/0.1% Tween 20. To improve access of antibodies to internal structures, the ventral skin was cut open. Larvae were incubated in blocking buffer (1% DMSO, 1% sheep serum, 1% BSA, 1% Triton X-100 in 1× PBS) for at least 60 min at room temperature. Samples were then incubated overnight at 4°C with primary antibody, washed and then reblocked and incubated in secondary antibody overnight at 4°C. Primary antibodies were: mouse anti-GFP (Roche, 11814460001, 1:100), mouse anti-glucagon (Sigma, G2654, 1:100), mouse anti-2F11 (Abcam, ab71286, 1:100), rabbit anti-somatostatin (Dako, A0566, 1:200), guinea pig anti-insulin (Dako, A0564, 1:200), rabbit anti-GFP (antikoerper, ABIN110592, 1:200) and rabbit anti-dsRed (Clontech, 632496, 1:200).

Secondary antibodies were labeled with Alexa Fluor (Invitrogen) and diluted 1:1000. Nuclei were labeled by incubation overnight at 4°C in 100 ng/ml DAPI/1% DMSO in PBS. Phalloidin staining (Alexa Fluor 488 Phalloidin, ThermoFisher, A12379) was performed as described (Goody et al., 2012).

### Microscopy

Live samples were mounted in 1.2% low melt agarose and overlaid with egg water or E3 medium containing 0.003% tricaine. Brightfield and low-magnification fluorescent images were captured on a Leica DM6000B. Imaging of fixed and live samples was performed using a Leica Sp5 laser scanning confocal microscope with a 63× glycerol-immersion objective or a Zeiss Axio Observer.Z1 equipped with a CSU-X1 spinning disc confocal using 25×, 40× or 63× water-immersion lenses. Time-lapse imaging of cell morphology was performed on a Leica Sp5 using a 40× air objective. Time-lapse images of cell protrusion dynamics were acquired using a microlens-enhanced Nipkow disk-based UltraVIEW RS confocal system (PerkinElmer) and a 40× water objective. Confocal imaging for islet assembly studies was performed using a Zeiss LSM5 with a 20× dipping lens or a Leica Sp5 with a 20× air objective. The HyD detector was used for optimal detection of the GFP signal on the Leica Sp5. For single-cell imaging, cell location was confirmed by first imaging memGFP (*mnx1:memGFP*) in combination with LifeActTom (*krt18:LifeActTom*), then the GFP channel was captured alone to maximize imaging speed. Details of acquisition settings for time-lapse studies are provided in Table S8. Further details of time-lapse imaging, cell and filopodia tracking are provided in the supplementary Materials and Methods.

### Image analysis

Confocal image stacks were processed using ImageJ. A median filter was used to reduce speckle noise; contrast adjustment and background subtraction were uniformly applied. Sample shift in time-lapse series was corrected using StackReg (http://bigwww.epfl.ch/thevenaz/stackreg/) (Thevenaz et al., 1998) and PoorMan3Dreg (developed by Michael Liebling; http://sybil.ece.ucsb.edu/pages/poorman3dreg/index.html) plug-ins of ImageJ. Sample shift occurring between acquisition of channels was corrected on *z*-projections using Photoshop (Adobe). Three-dimensional (3D) visualizations were prepared using Imaris (Bitplane). Cell counts based on transgene expression were performed using the Spot Detection function of Imaris with a spot diameter of 5 µm by 3D visualization of a confocal *z*-stack spanning the region. Objects were filtered based on signal intensity at the center. Cell counting based on immunohistochemistry with DAPI staining was achieved using the Point Picker plug-in of ImageJ. Cell counting results are representative of two independent experiments.

#### Islet quantitation

For islet size determination using Imaris, the Surfaces function was applied. Images were processed by smoothing (0.5 µm) and local background subtraction, then contour surfaces were generated that enclosed the GFP (*pax6b:GFP*) fluorescent signal in the pancreatic tail, and volumes were determined. Signal threshold and object size filtering were applied consistently to all images. For counting GFP^+^/RFP^+^ cells, the previously determined surfaces were applied to mask signals external to the secondary islets. The masked RFP^+^ nuclei were counted as previously described. 3D rendering was performed using the Quick 3D function of Imaris. 3D islet volume analysis procedures using the Particle Analyser plug-in (Doube et al., 2010) of ImageJ are detailed in the supplementary Materials and Methods. Image acquisition and analysis parameters were optimized within each experiment. Reported differences between control and treated groups were consistent in two or more independent experiments.

#### Automated islet quantitation

For the automated detection of islet volumes, a custom feature detection pipeline was developed that is adequate for batch processing large numbers of images. It was implemented in the Quocmesh library (A. G. Rumpf, Institute for Numerical Simulation, University of Bonn, Germany), using C++ for image processing, and Linux shell scripts for file management and automation. Our custom script requires an initial user interaction step to delineate the boundary between the pancreatic head region and tail, then subsequent delineation of the pancreas based on mCherry (*ela:mCherry*) expression and volume quantitation of GFP^+^ (*pax6b:GFP*) secondary islets proceeds without further intervention (Fig. 6B). Imaging settings and processing parameters were adjusted for each experiment for optimal detection of true objects. The chosen settings were applied uniformly. Details of the processing pipeline are provided in the supplementary Materials and Methods. When processing is completed, a table is generated containing islet volumes, and tiled image montages show pre- and post-processing images for an overview and visual assessment of the analysis. Results shown are representative of at least two independent experiments.

### Statistical analysis

Data were analyzed and graphs were produced using Prism (GraphPad). Statistical tests were applied as indicated in the results and figure legends.

## Supplementary Material

Supplementary information

Supplementary information
