# Supplementary material for: In vivo imaging of emerging endocrine cells reveals a requirement for PI3K-regulated motility in pancreatic islet morphogenesis
Source: Development. 2018 Feb 1;145(3):dev158477. doi: 10.1242/dev.158477 (PMC5818004; doi:10.1242/dev.158477)
Supplement: Supplementary information [file develop-145-158477-s1.pdf]

Supplementary Methods

DNA Constructs and Generation of transgenic lines

To generate *krt18:LifeActTom*, 3.9kb upstream of *keratin 18* was amplified from genomic DNA of 24 hpf embryos and cloned into the 5' Gateway entry vector (*p5E-krt18Pr*). The following primers were used:

For: AGGACATCTGCCCTCCAGCAC
Rev: GTCGCTGGTGTAAGTGAGCAGACG

The Tandem-dimer-Tomato (Tom) fluorophore was PCR amplified from *pCAG-2A-H2B-Tomato* (gift of H. Lickert), fused downstream of the LifeAct peptide in *pGEM-T-LifeAct* (gift of R. Aufschnaiter), then cloned into the Gateway middle entry vector (*pME-LifeActTom*). The final construct, *krt18:LifeActTom*, was generated by a Gateway reaction (Kwan et al., 2007). For generating *hsp70:LifeActTom* by Gateway cloning, the *hsp70* promoter in *p5E-hsp70* (Kwan et al., 2007) was combined with *pME-LifeActTom* in the *pDestTol2pA* destination vector. *hsp70:LifeActTom-PTX* was assembled by Gateway cloning in the *pDestTol2CG2* destination vector (Kwan et al., 2007), which contains the *cmlc2:GFP* transgenesis marker. A middle entry vector (*pME-LifeActTom-PTX*) was created containing the coding sequence for *PTX* (gift of A. Gavales) downstream of *LifeActTom*, with an intervening 2A sequence to drive separate expression of the two proteins. The *ela:mCherry* line was generated by Gateway cloning of the following entry vectors: *p5E-elastase* promoter, containing 1.9kB of the *elastase3l* promoter (Wan et al., 2006), *pME-mCherry* (Kwan et al., 2007), *p3E-polyA* (Kwan et al., 2007) into the *pDestTol2CG2* destination vector (Kwan et al., 2007). The *ins:mKO2* construct was generated by cloning the *ins* promoter (Moro et al., 2009, gift of F. Argenton) upstream of *mKO2-zCdt1* (Sugiyama et al., 2009, gift of A. Miyawaki) in the *pT2KXIGΔin* backbone.

Plasmid DNA was injected in combination with transposase mRNA into one-cell stage mitfa embryos. Embryos (sorted for fluorescent signal where possible) were raised to adulthood and crossed to identify germ-line transmitting founders. For each construct, several transgenic lines showed similar expression patterns, the line with consistent strong expression was maintained and used for further experiments.

Time Lapse Imaging

Time lapse studies were initiated with a z-stack that extended beyond the sample in both

Development • Supplementary information

directions, to account for potential sample movement. Data was not included for analysis in cases in which the cell shifted out of view. When possible, the sample was re-positioned and imaging re-started. This causes the occasional appearance of irregular time intervals in our time lapse image sequences. A proportion of image series could not be analyzed due to poor quality (low signal or loss of signal over time) or difficulty in distinguishing individual cells. In some samples, signal recovery permitted resumption of analysis at subsequent time points, with times of image acquisition as indicated in the figures.

As older fish (>5dpf) show decreased viability following continuous imaging for more than 2-3h, imaging studies extending >3h were performed by adapting a 'catch-and-release' approach (Mumm et al., 2006). In brief, samples were mounted in low melt agarose in glass bottom plates (maximum 2 per plate), overlaid with tricaine and directly imaged. The x,y coordinates of the imaged region were recorded relative to the primary islet to enable identification of the position at later times. After imaging, samples were carefully removed from the agarose using fine forceps, rinsed in egg water, and returned to the incubator in individual 3cm plates until the next imaging session.

To follow clustering of Notch inhibitor-induced endocrine cells from 6-9 dpf (Fig. S2), 20 samples were imaged in total. 9/9 that were followed to 8 or 9 dpf showed cluster formation. 5/8 samples imaged to 7 dpf had formed clusters, while the remaining 3 showed cell movements but had not formed clusters. 3 samples were not analyzed as they did not survive beyond the first time point. Regions for extended imaging in uninduced 13-15 dpf samples were selected to contain single cells within 2-3 cell diameters of a larger cluster or of other cells. Already formed clusters or isolated single cells were not followed.

Cell Tracking

For analysis of time lapse series, image stacks were first cropped and registered using ImageJ. Images for extended time series were manually aligned. Individual cells followed over time in z-projections were highlighted by a color overlay using Photoshop. Cells were manually tracked using 3D visualization in Imaris, with identity based on relative positions

Development • Supplementary information

and relation to nearby structures. In some series, cells could be followed based on GFP in addition to dsRed expression. A subset of dsRed$^+$ cells became GFP$^+$ over time, reflecting progressive differentiation of endocrine progenitor into beta cells. To quantitate cell clustering, cell coordinates (x,y,z) at each time point were determined using Imaris and exported to Matlab. Volume of a polygon that contains the tracked cells was calculated using the 'convex hull' function of Matlab. Polygon representations were generated in Matlab.

Filopodia Tracking

For filopodia tracking, the image stack was processed using ImageJ. The image was first cropped to contain a single cell. Contrast enhancement and background subtraction were performed, and gamma adjustment was applied to enhance the weak signal of fine protrusions. Filopodia length was measured in each frame using the 'Neuron Growth' plugin for ImageJ (Fanti et al., 2011). As recommended (Fanti et al., 2011), images were resized twofold to increase pixel density and improve detection of boundary features. Detection was performed using the automatic mode where possible, with manual correction applied as necessary. Cell morphology and motility were analyzed on 2D projections, recognizing that information along the z-axis will go undetected. From a lateral view, the pancreas extends primarily along the x-y dimension. Z-projections were necessary in order to have sufficient signal intensity to detect boundaries and fine protrusions in the x-y dimension. Of 13 time lapse movies acquired in 2 independent experiments, 5 had cells with robust membrane signal, and maintained signal intensity that was sufficient for filopodial tracking analysis. In all, 41 filopodia were analyzed.

Quantitation of cell morphology and membrane dynamics

For analysis of morphology and membrane dynamics in time series, PI3K-inhibitor treated and control samples at 6 dpf were imaged at 3-minute intervals with 1024x1024 pixel resolution. For analysis of cell morphology in *hsp:LifeActTom-PTX* transgenics and controls, heat-shocked samples at 7 dpf were imaged at 4-minute intervals with 512x512 pixel resolution to minimize bleaching, and images were resized to double the pixel density. Images were smoothed by a median filter, and gamma adjustment was applied. Cell morphology analysis was performed using the ImageJ plugin ADAPT (Barry et al., 2015) on image series cropped to contain a single cell. Parameters were adjusted empirically for optimized detection. Fine protrusions not recognized automatically were outlined manually.

Development • Supplementary information

Cell circularity is calculated as 4π(area/perimeter^2). Solidity is the area divided by the area of the convex hull (Fig. S8A). Membrane dynamics were determined using CellGeo (Tsygankov et al., 2014), using data sets of cell boundaries computed by the ADAPT PlugIn. Total cell membrane dynamic activity (protrusion + retraction) between successive video frames was normalized to cell perimeter. The boundary velocity threshold was set to 5.

Secondary islet quantitation

In control experiments, islet formation was most robust and consistent when more cells were induced. In a previous report, maximal islet cell induction was achieved with 50μM Ly411575 for up to 3 days (Ninov et al., 2012). For our islet assembly assay, we applied an induction method using lower doses of notch inhibitor and retinoic acid inhibitior for 24h, as samples are subjected to an additional 48h of inhibitor treatments. Combining low-dose inducing treatments acting on 2 different pathways minimized systemic toxicity, and represents the milieu that controls physiological islet cell differentiation (Huang et al., 2014; Kimmel and Meyer, 2016). Results shown for islet assembly assays are representative of at least 2 independent experiments.

To quantitate inhibitor effects on secondary islet formation, 3D object analysis was performed using the Particle Analyzer of ImageJ (Doube et al., 2010). Image stacks were prepared for analysis by preprocessing steps using ImageJ, in which the pancreas is manually outlined based on mCherry expression in the exocrine pancreas, to define the location of the secondary islets, and exclude other GFP$^+$ cells in the region (Fig. S10B-C, for details see Supplemental Protocol 1, below). For experiments using heat-shock inducible PTX expression, in preprocessing steps the pancreas is manually outlined based on a corresponding brightfield image, followed by analysis using the Particle Analyzer (see Supplemental Protocol 2, below).

Automated islet quantitation

The process for automated analysis of islet volumes involved the following steps: (1) The user is presented with an image combining the middle slice of the red channel (exocrine pancreas) and a maximum-intensity projection of the green channel (endocrine islets), and required to mark a line separating the pancreatic head from the tail region. (2) Both channels are blended with a histogram equalized version of themselves, to achieve contrast enhancement. (3) The red channel (pancreas) is segmented using the Chan-Vese model

Development • Supplementary information

(Chan and Vese, 2001), with an additional term added for boundary avoidance. (4) The image for volume detection is prepared by first excluding points outside the segmented pancreas or anterior to the pancreatic tail, then the intensity and gradient of the green image are combined. To reduce the sensitivity to noise, the image gradients are computed by applying an anisotropic Gaussian filter. (5) Object boundaries are determined by thresholding the image of the previous step and triangulating with standard level set techniques (Sethian, 1999). (6) An edge connectivity measure is applied to split minimally connected objects, and hidden components (ie, completely contained inside others) are discarded. (7) Finally, the volume and surface area of each component is computed. Minimum object size is 50μm^3. In addition, we apply the assumption that islets have a certain regularity of shape, and components with isoperimetric ratio (Area/Volume^(2/3)) above a defined threshold were considered noise and discarded. (Details of program code used for analysis are available upon request.)

Development • Supplementary information

Supplemental Protocol 1

Software Tools required:
ImageJ - https://imagej.nih.gov/ij/download.html
StackReg PlugIn – http://bigwww.epfl.ch/thevenaz/stackreg (Thevenaz et al., 1998)
Particle Analyser PlugIn– http://bonej.org/particles (Doube et al., 2010)

File requirements: 2 channel image, endocrine pancreas/exocrine pancreas

- Open image
- If necessary depending on file type and import method:

  [Image > Properties > Assign pixel size (width, height, depth)]

- Split Channels: Image > Color > Split channels
- Select channel with islets
- Smooth image: Process > Filters > Median (radius = 1)
- Enhance Contrast: Process > Enhance Contrast (saturated=0.4, equalize, process all slices)
- If gut movements caused shift during image acquisition: apply StackReg (Rigid Body)

[Additional pre-processing, such as background subtraction, can further improve islet detection (apply uniformly to data set).]

- Perform Z projection of islets: Image > Stack > Z-project (Max Intensity)
- Select image stack of *ela:mCherry*$^{+}$ exocrine pancreas
- Smooth image: Process > Filters > Median (radius = 2, process all slices)
- Perform Z projection of *ela:mCherry* image: Image > Stack > Z-project (Max Intensity)
- Set tool to ‘polygon’ and outline pancreas as a closed object
- Select projection of pancreatic islets
- Apply pancreas outline to islets : Edit > Selection > Restore Selection
- Adjust selection to include only posterior pancreas
- Select stack of pancreatic islets
- Apply polygon to define posterior islets: Edit > Selection > Restore Selection
- Clear outside – Edit > Clear outside (Stack)
- Set threshold: Image > Adjust > Threshold (apply uniformly across experimental data set)
- Volume analysis: “Particle Analyser” (min = 100(50)*; max=100000; other settings default)
- Save results table.

→ * Value depends on imaging conditions, can decrease to 50 if robust signal with high signal-to-noise ratio.

Development • Supplementary information

Supplemental Protocol 2

Software Tools required:

ImageJ - https://imagej.nih.gov/ij/download.html

StackReg PlugIn – http://bigwww.epfl.ch/thevenaz/stackreg (Thevenaz et al., 1998)

Particle Analyser PlugIn– http://bonej.org/particles (Doube et al., 2010)

File requirements: image stack of endocrine pancreas and corresponding bright field

- Open image
- If necessary depending on file type:

  [Image > Properties > Assign pixel size (width, height, depth)]

- Split Channels: Image > Color > Split channels
- Select channel with islets
- Smooth image: Process > Filters > Median (radius = 1)
- If gut movements caused shift during image acquisition: apply StackReg (Rigid Body)
- Perform Z projection of islets: Image > Stack > Z-project (Max Intensity)
- Select brightfield image – choose z-slice where pancreas can be best identified
- Set tool to 'polygon' and outline pancreas as a closed object
- Select <u>projection</u> of pancreatic islets
- Apply pancreas outline to islets : Edit > Selection > Restore Selection
- Adjust selection to include only posterior pancreas
- Select <u>stack</u> of pancreatic islets
- Apply polygon to define posterior islets: Edit > Selection > Restore Selection
- Clear outside – Edit > Clear outside (Apply to all slices)
- Set threshold: Image > Adjust > Threshold (apply uniformly across experimental data set)
- Volume analysis: "Particle Analyser" (min = 100*; max=100000; other settings default)
- Save results table.

Development • Supplementary information

Supplementary References

Barry, D.J., Durkin, C.H., Abella, J.V., and Way, M. (2015). Open source software for quantification of cell migration, protrusions, and fluorescence intensities. *The Journal of cell biology* **209**, 163-180.

Chan, T.F., and Vese, L.A. (2001). Active contours without edges. *IEEE transactions on image processing : a publication of the IEEE Signal Processing Society* **10**, 266-277.

Delporte, F.M., Pasque, V., Devos, N., Manfroid, I., Voz, M.L., Motte, P., Biemar, F., Martial, J.A., and Peers, B. (2008). Expression of zebrafish pax6b in pancreas is regulated by two enhancers containing highly conserved cis-elements bound by PDX1, PBX and PREP factors. *BMC developmental biology* **8**, 53.

Doube, M., Klosowski, M.M., Arganda-Carreras, I., Cordelieres, F.P., Dougherty, R.P., Jackson, J.S., Schmid, B., Hutchinson, J.R., and Shefelbine, S.J. (2010). BoneJ: Free and extensible bone image analysis in ImageJ. *Bone* **47**, 1076-1079.

Fanti, Z., Martinez-Perez, M.E., and De-Miguel, F.F. (2011). NeuronGrowth, a software for automatic quantification of neurite and filopodial dynamics from time-lapse sequences of digital images. *Dev Neurobiol* **71**, 870-881.

Flanagan-Steet, H., Fox, M.A., Meyer, D., and Sanes, J.R. (2005). Neuromuscular synapses can form in vivo by incorporation of initially aneural postsynaptic specializations. *Development* **132**, 4471-4481.

Huang, W., Wang, G., Delaspre, F., Vitery Mdel, C., Beer, R.L., and Parsons, M.J. (2014). Retinoic acid plays an evolutionarily conserved and biphasic role in pancreas development. *Dev Biol* **394**, 83-93.

Kimmel, R.A., and Meyer, D. (2016). Zebrafish pancreas as a model for development and disease. *Methods Cell Biol* **134**, 431-461.

Kwan, K.M., Fujimoto, E., Grabher, C., Mangum, B.D., Hardy, M.E., Campbell, D.S., Parant, J.M., Yost, H.J., Kanki, J.P., and Chien, C.B. (2007). The Tol2kit: a multisite gateway-based construction kit for Tol2 transposon transgenesis constructs. *Dev Dyn* **236**, 3088-3099.

Moro, E., Gnugge, L., Braghetta, P., Bortolussi, M., and Argenton, F. (2009). Analysis of beta cell proliferation dynamics in zebrafish. *Dev Biol* **332**, 299-308.

Mumm, J.S., Williams, P.R., Godinho, L., Koerber, A., Pittman, A.J., Roeser, T., Chien, C.B., Baier, H., and Wong, R.O. (2006). In vivo imaging reveals dendritic targeting of laminated afferents by zebrafish retinal ganglion cells. *Neuron* **52**, 609-621.

Ninov, N., Borius, M., and Stainier, D.Y. (2012). Different levels of Notch signaling regulate quiescence, renewal and differentiation in pancreatic endocrine progenitors. *Development* **139**, 1557-1567.

Obholzer, N., Wolfson, S., Trapani, J.G., Mo, W., Nechiporuk, A., Busch-Nentwich, E., Seiler, C., Sidi, S., Sollner, C., Duncan, R.N.*, et al.* (2008). Vesicular glutamate transporter 3 is required for synaptic transmission in zebrafish hair cells. *J Neurosci* **28**, 2110-2118.

Parsons, M.J., Pisharath, H., Yusuff, S., Moore, J.C., Siekmann, A.F., Lawson, N., and Leach, S.D. (2009). Notch-responsive cells initiate the secondary transition in larval zebrafish pancreas. *Mech Dev* **126**, 898-912.

Pisharath, H., Rhee, J.M., Swanson, M.A., Leach, S.D., and Parsons, M.J. (2007). Targeted ablation of beta cells in the embryonic zebrafish pancreas using E. coli nitroreductase. *Mech Dev* **124**, 218-229.

Preibisch, S., Saalfeld, S., and Tomancak, P. (2009). Globally optimal stitching of tiled 3D microscopic image acquisitions. *Bioinformatics* **25**, 1463-1465.

Sethian, J.A. (1999). Level set methods and fast marching methods. In Cambridge Monographs on Applied and Computational Mathematics (Cambridge, Cambridge University Press).

Sugiyama, M., Sakaue-Sawano, A., Iimura, T., Fukami, K., Kitaguchi, T., Kawakami, K., Okamoto, H., Higashijima, S., and Miyawaki, A. (2009). Illuminating cell-cycle progression in the developing zebrafish embryo. *Proc Natl Acad Sci U S A* **106**, 20812-20817.

Thevenaz, P., Ruttimann, U.E., and Unser, M. (1998). A pyramid approach to subpixel registration based on intensity. *IEEE transactions on image processing : a publication of the IEEE Signal Processing Society* **7**, 27-41.

Tsygankov, D., Bilancia, C.G., Vitriol, E.A., Hahn, K.M., Peifer, M., and Elston, T.C. (2014). CellGeo: a computational platform for the analysis of shape changes in cells with complex geometries. *The Journal of cell biology* **204**, 443-460.

Wan, H., Korzh, S., Li, Z., Mudumana, S.P., Korzh, V., Jiang, Y.J., Lin, S., and Gong, Z. (2006). Analyses of pancreas development by generation of gfp transgenic zebrafish using an exocrine pancreas-specific elastaseA gene promoter. *Exp Cell Res* **312**, 1526-1539.

Zecchin, E., Filippi, A., Biemar, F., Tiso, N., Pauls, S., Ellertsdottir, E., Gnugge, L., Bortolussi, M., Driever, W., and Argenton, F. (2007). Distinct delta and jagged genes control sequential segregation of pancreatic cell types from precursor pools in zebrafish. *Dev Biol* **301**, 192-204.

Development • Supplementary information

Supplementary Figures

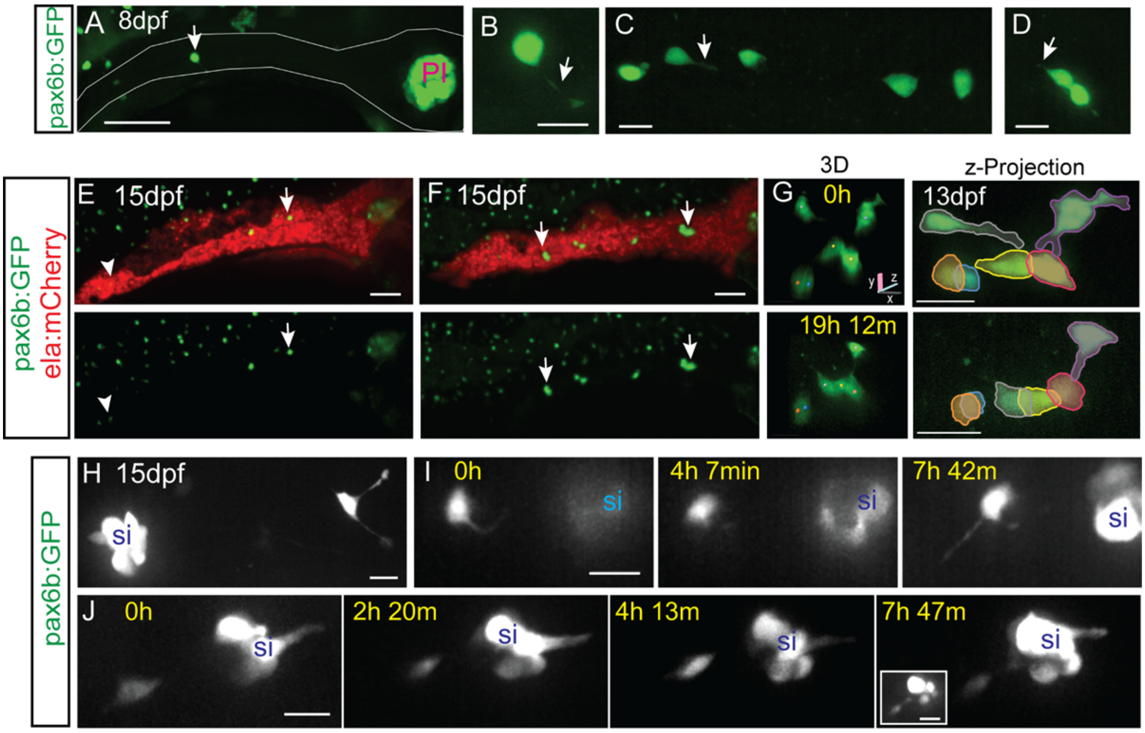

Figure S1. Morphology and dynamics of naturally occurring secondary islet cells. Related to figure 1.

(A) Maximal projection of confocal stack of *pax6b:dsRed* transgenic at 8 dpf. Scale bar = 50µm. (B) Close-up view of endocrine cell (in A, arrow), which displays a long protrusion (B, arrow). (C, D) Close-up views of secondary endocrine cells in samples as in A. Arrows indicate cell protrusions. (B-D) Scale bars = 10µm. (E, F) Maximal projections of confocal stacks of *pax6b:dsRed;ela:mCherry* transgenic at 15 dpf. Larvae show variable islet development, ranging from small islets (arrow, E) and single cells (arrowhead, E), to larger clusters (arrows, F). Scale bar = 50µm. (G) Islet cells at times indicated in 13 dpf *pax6b:GFP* transgenic. 3D view (left) showing tracked cells (colored spheres), maximal projection (right) with cells pseudocolored to highlight cell movements. Single time point (H) and image series at times indicated (I-J) from 15 dpf *pax6b:GFP* transgenics. Single cells show protrusions (H, I) and move closer to existing secondary islets (I, J). Increasing contrast reveals cell protrusions (J, right, inset). PI, principal islet; si, secondary islet. (G-J) Scale bar = 10µm. (For sample details see Tables S1, S2, S3.)

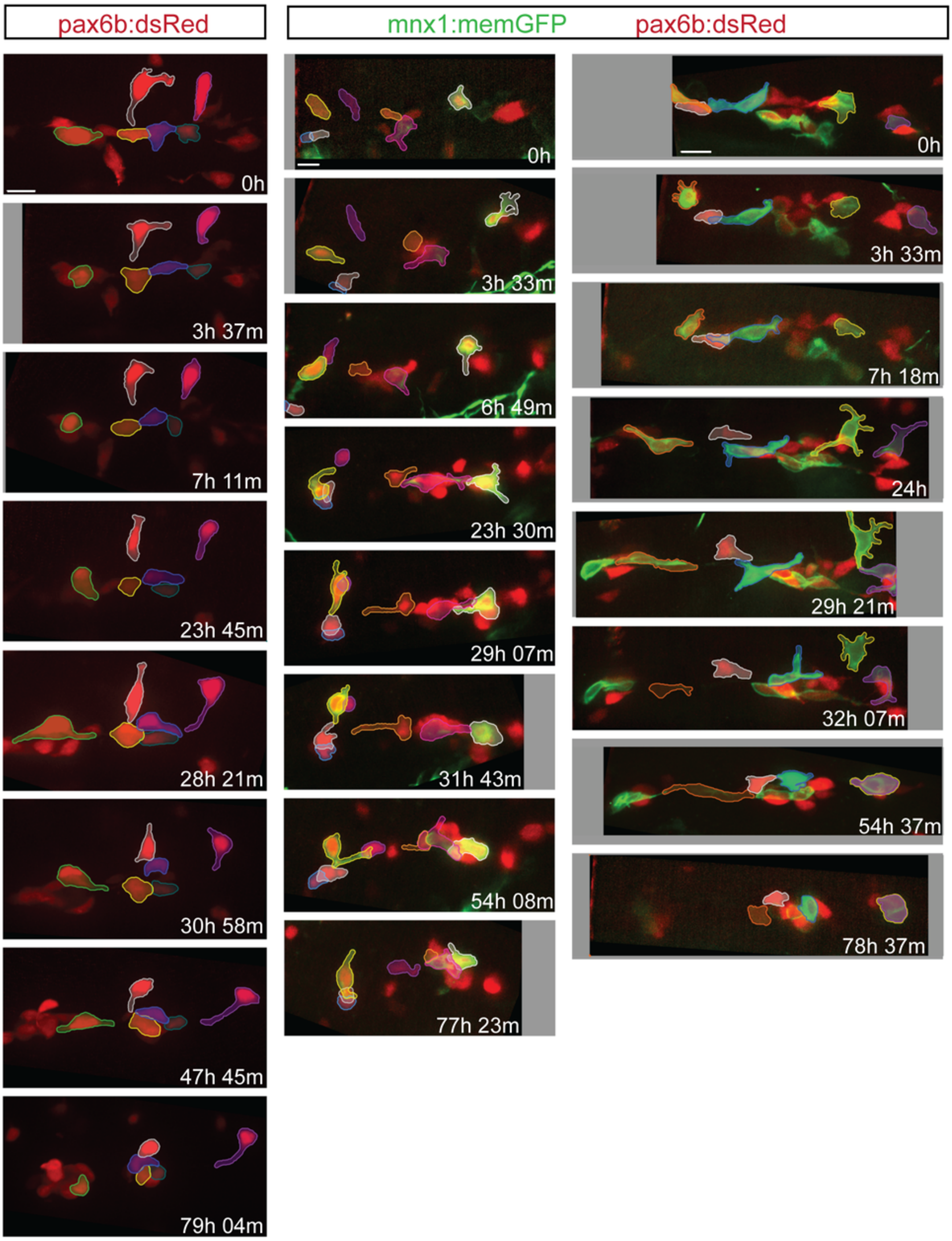

Fig S2. Induced endocrine cells cluster over ≥72 hours.

Islet morphogenesis was followed in *pax6b:dsRed* (left) and *pax6b:dsRed;mnx1:memGFP* (center, right) transgenics beginning at 6dpf, by a modified catch-and-release approach (see Supplementary Methods), following Notch inhibitor treatment at 4 dpf for 24 hours. Selected cells are labeled by pseudocoloring to facilitate identification in subsequent images. Scale bars = 10μm. Sample details are described in Supplementary Methods.

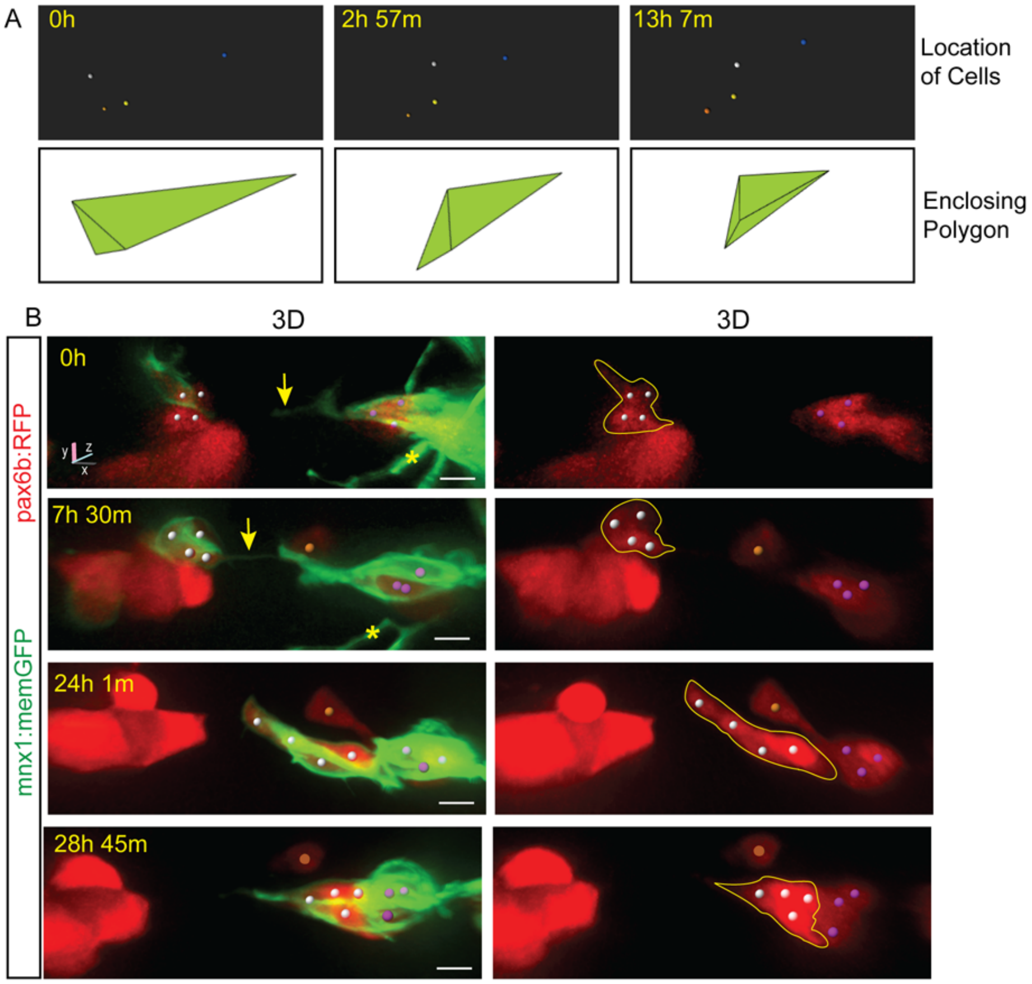

Fig S3. Protrusion formation coincides with endocrine cell movements and clustering. Related to Figure 2.

(A, top) Tracked cells (colored spheres) shown alone, from the time series shown in Fig 2B. (A, bottom) Enclosing polygon as calculated using Matlab. Polygon volume reflects the spread of cells in three-dimensional space, and thus provides an index of cell clustering. (B) Image series of pancreatic endocrine cells in *pax6b:dsRed;mnx1:memGFP* transgenics following Notch inhibitor treatment from 4 dpf to 5 dpf, and imaged at 6 dpf. 3D representations with cells tracked (colored spheres) using Imaris. Movement of cells follows the appearance of a fine intercellular tether (arrow). Scale bars = 10μm. *Indicates neurite projections from *mnx1:memGFP* transgene expressed in overlying neurons.

Development • Supplementary information

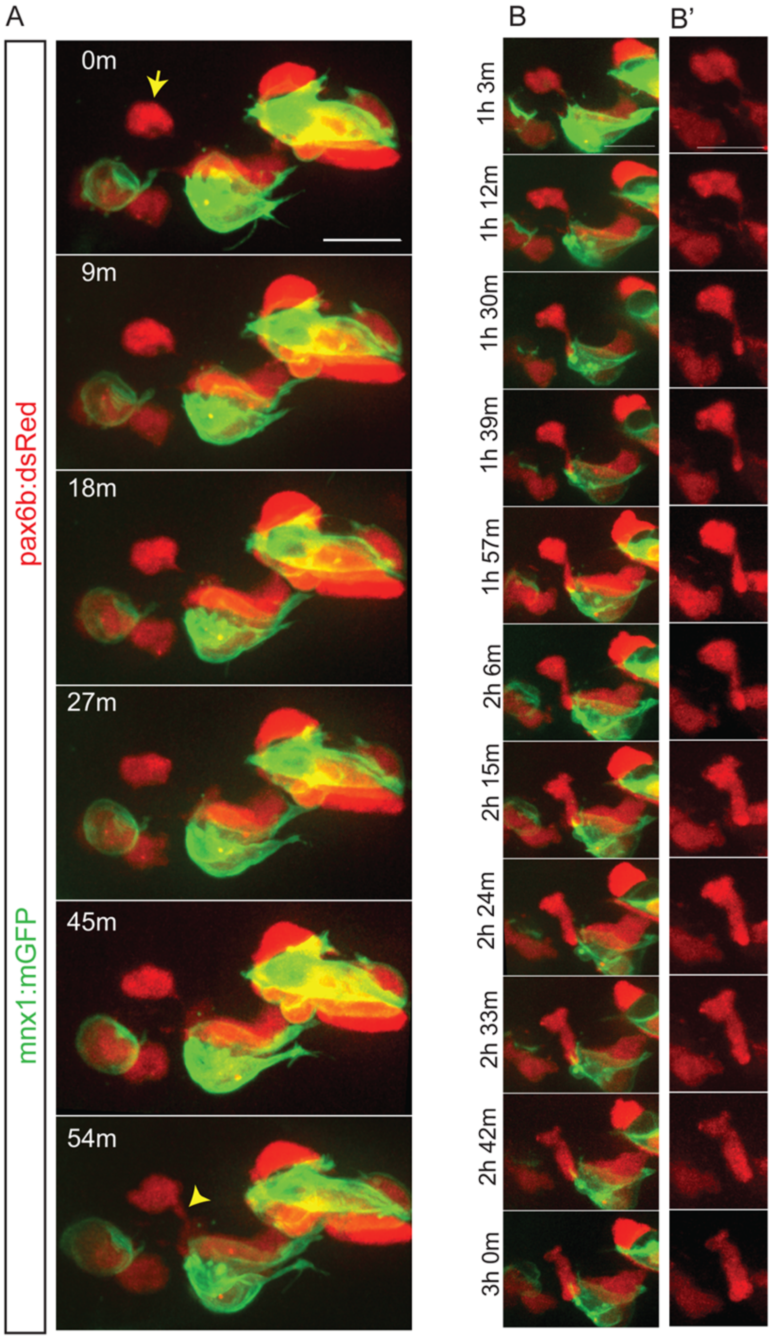

Fig S4. Dynamics of cell coalescence. Related to Figure 3.

Z-projections from time-lapse image series of pancreatic endocrine cells in *pax6b:dsRed;mnx1:memGFP* transgenics following Notch inhibitor treatment at 4 dpf. Sample was imaged at 7 dpf, with images acquired every 9 minutes. (A) The indicated cell (arrow) forms a connection to a nearby cluster which strengthens over time (bottom frame, arrowhead). (B) Further time points of the sample shown in (A), projections of a subset of z-slices to highlight cell shape changes that accompany coalescence of a single cell with a cluster. (B') dsRed channel shown alone for clarity. Scale bars = 10μm.

Development • Supplementary information

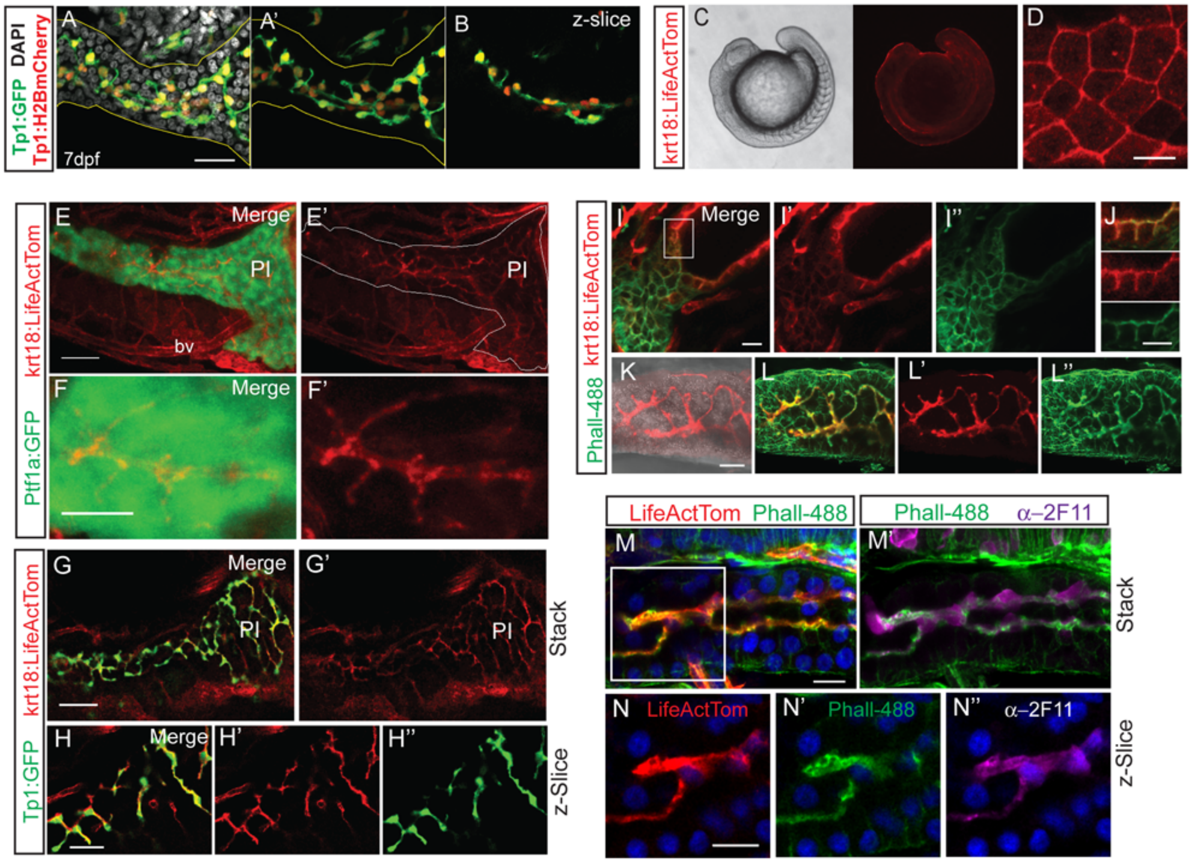

Figure S5. Pancreatic duct morphology delineated with *krt18:LifeActTom* transgene.

(A) Maximal projection of confocal z-stack showing pancreatic duct morphology at 7 dpf delineated by expression of *Tp1:H2BmCherry* (red, nuclear) and *Tp1:GFP* (green, cytoplasmic), immunostained for GFP. Nuclei (gray) are stained by DAPI. Pancreas is delineated by yellow outline. (A') Image as in (A) with DAPI signal removed. (B) Single z-slice of sample shown in (A). (C) Widefield view showing *krt18:LifeActTom* transgene expression in surface epithelium in 15-somite stage embryo (right), with corresponding brightfield view (left). (D) Maximum intensity projection of confocal stack of surface epithelium of embryo as in (C), showing actin accumulation at cell-cell junctions. Scale bar = 25μm. (E-F) Projection of confocal stack through the pancreas in *krt18:LifeActTom;ptf1a:GFP* larva at 6 dpf. (E'-F') *krt18:LifeActTom* expression, with GFP channel removed. (E') Pancreas as defined by *ptf1a:GFP*, is outlined in white. LifeActTom is also expressed in some blood vessels (bv). PI = principal islet. (E) Scale bar = 50μm. (F) Scale bar = 15μm. (G-G') Projected substack within the pancreas of *krt18:LifeActTom;Tp1:GFP* larva at 6dpf. Scale bar = 15μm. (H-H") Single z-plane of pancreas as in (G). Merged image (H) and corresponding single channel images (H', H"). (I)

Development • Supplementary information

Projection of confocal stack of 6 dpf *krt18:LifeActTom* larva, co-labeled with Phalloidin-488 (Phall-488, green). (J) Single slice merged image (top) and single channels (middle, bottom), of the region boxed in (I), showing actin labeling of apical membranes within the developing gut epithelium. Scale bar = 10µm. (K) *krt18:LifeActTom* labels intrapancreatic duct, shown is a projected z-stack, overlay with brightfield image. (L) Pancreatic expression of *krt18:LifeActTom* overlaps with actin-rich duct structures highlighted by Phall-488. Merged image (L) and corresponding single channel images (L', L''). (M) 6 dpf *krt18:LifeActTom* larva labeled with anti-dsRed antibody and Phall-488, nuclei are labeled with DAPI. (M') Same sample as in (M), immunostained to indicate localization of Phall-488 in relation to 2F11 antibody. (N, N', N'') Single channel, single z-plane images of the region indicated in M (white box).

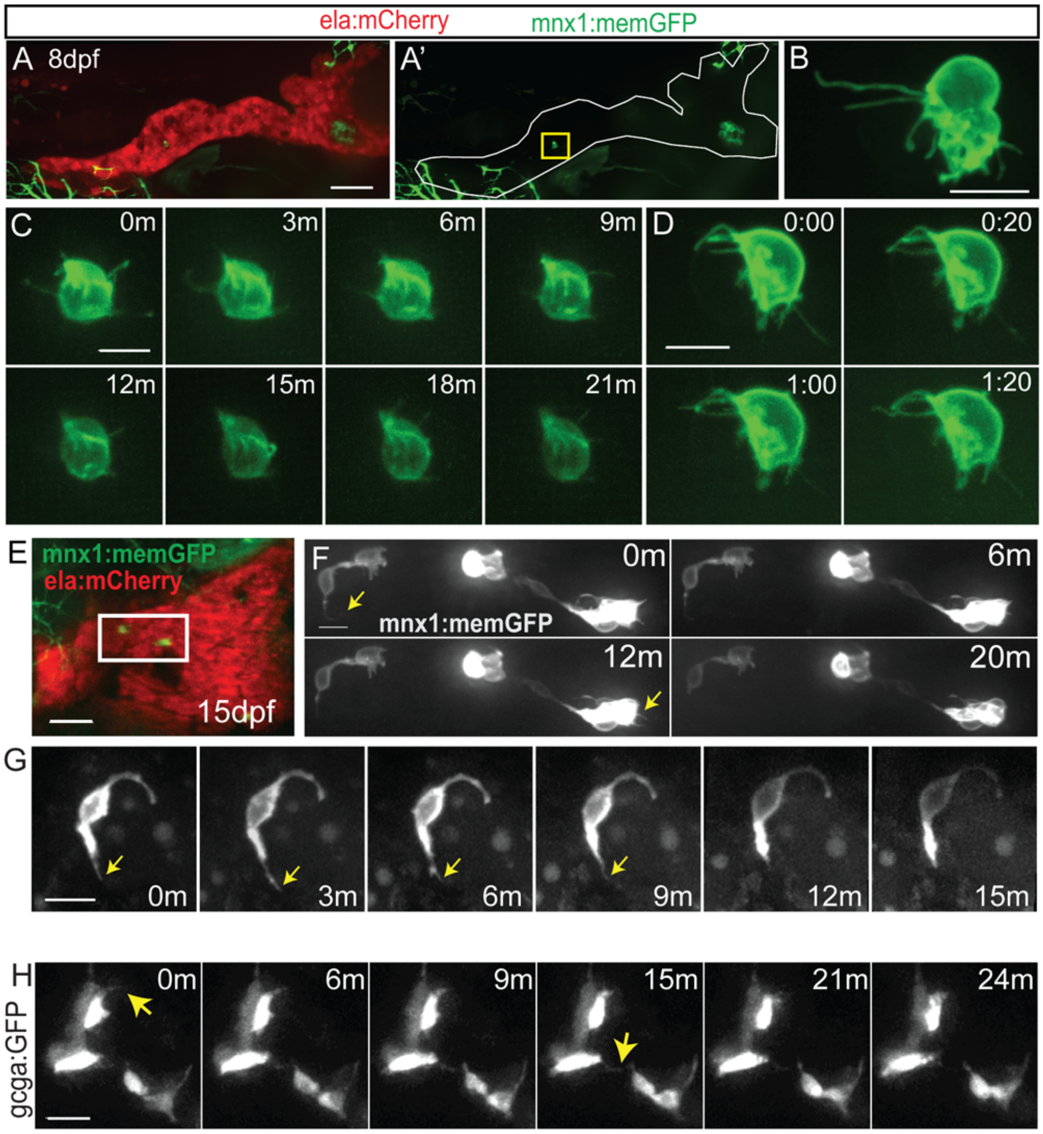

Figure S6. Protrusions in naturally occuring secondary islet cells.

(A) Maximal projection of confocal stack of *mnx1:memGFP;ela:mCherry* transgenic at 8 dpf. *mnx1:memGFP*-positive cells are rare at this stage (see Table S1). (A') GFP signal shown alone for clarity, a single cell is indicated (yellow box). Scale bar = 50μm. This image was assembled by stitching together images of partially overlapping regions, using the Pairwise Stitching Plugin for ImageJ (Preibisch et al., 2009). (B) Close-up view of endocrine cells showing long protrusions, from larva as in (A). Scale bar = 10μm. (C, D) Time lapse series of cells as in sample (A), at the times indicated in minutes (C) and min:sec (D). Extension and retraction of fine protrusions can be observed. (E) At 15 dpf, *mnx1:memGFP*-positive cells are more frequently observed, and small clusters can be detected (white box). Scale bar = 50μm. (F) Time lapse series of cells from sample in (E), which display fine dynamic protrusions. Nonlinear gamma adjustment was applied to enhance weak signals of

Development • Supplementary information

protrusions. Scale bar = 10µm. (G) Time lapse series of cell as in sample (E), at the times indicated in minutes (m). Arrow indicates protrusion. (H) Confocal projections from time lapse series of 7 dpf *gcga:GFP* transgenic that was treated with Notch inhibitor at 4 dpf. Nonlinear gamma adjustment was applied to enhance weak contrast signals. Arrows indicate dynamic protrusions. Scale bar = 10µm.

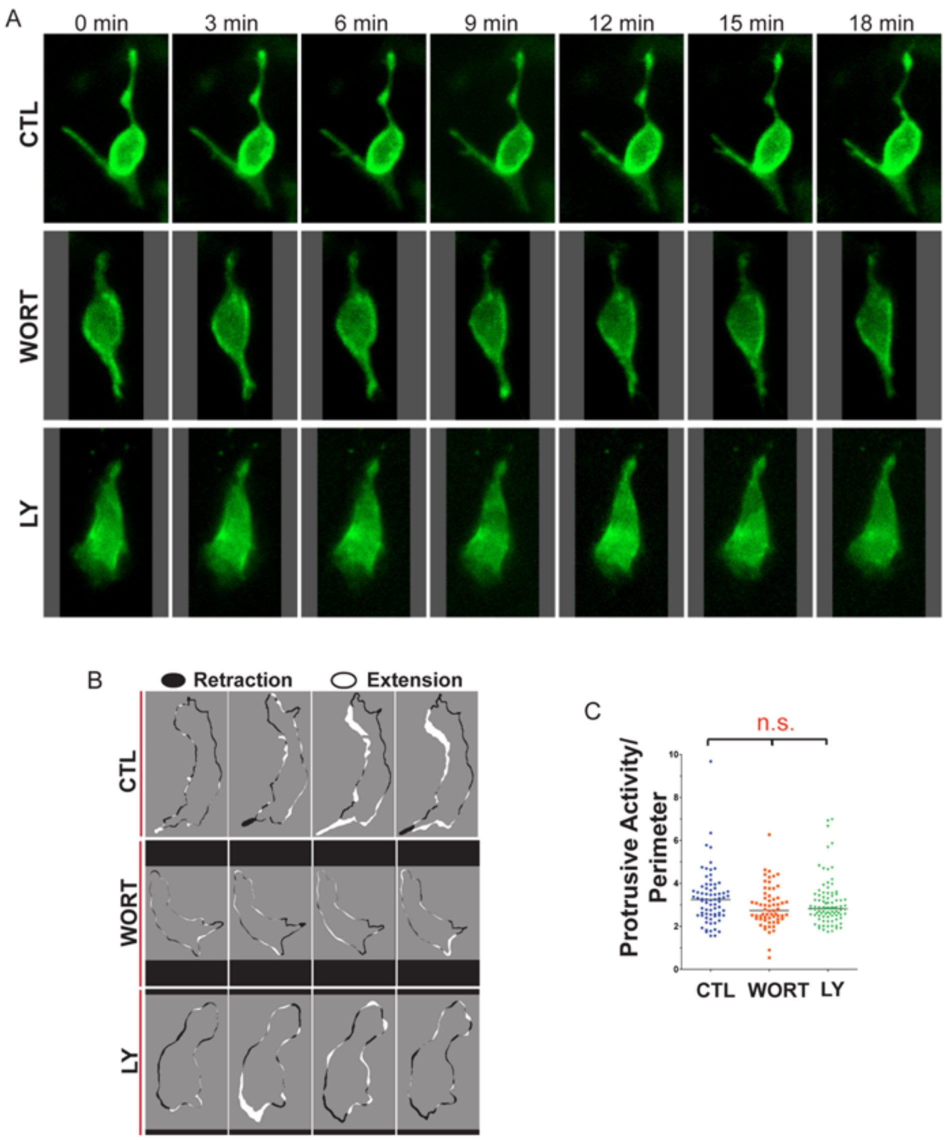

Figure S7. Single-cell analysis of endocrine cell dynamics. Related to Figure 5.

(A) Time series of representative single cells, in *mnx1:memGFP* transgenics treated to induce secondary islets from 4-5 dpf, followed by treatment at 6 dpf as indicated (WORT, 100nM wortmannin, LY, 50μm Ly294002 or CTL, DMSO), for 3-4 hours prior to imaging. (B) Membrane protrusion analysis indicates regions of expansion (white) and retraction (black) around the cell perimeter between adjacent frames in time lapse series of control, WORT, and LY treated embryos, imaged as in (A). (C) Protrusive Activity (area of expansion + area of retraction) for each cell over time, analyzed as in (B), normalized to cell perimeter. (n.s., not significant; Kruskal-Wallis followed by Dunn's Multiple Comparison test).

Development • Supplementary information

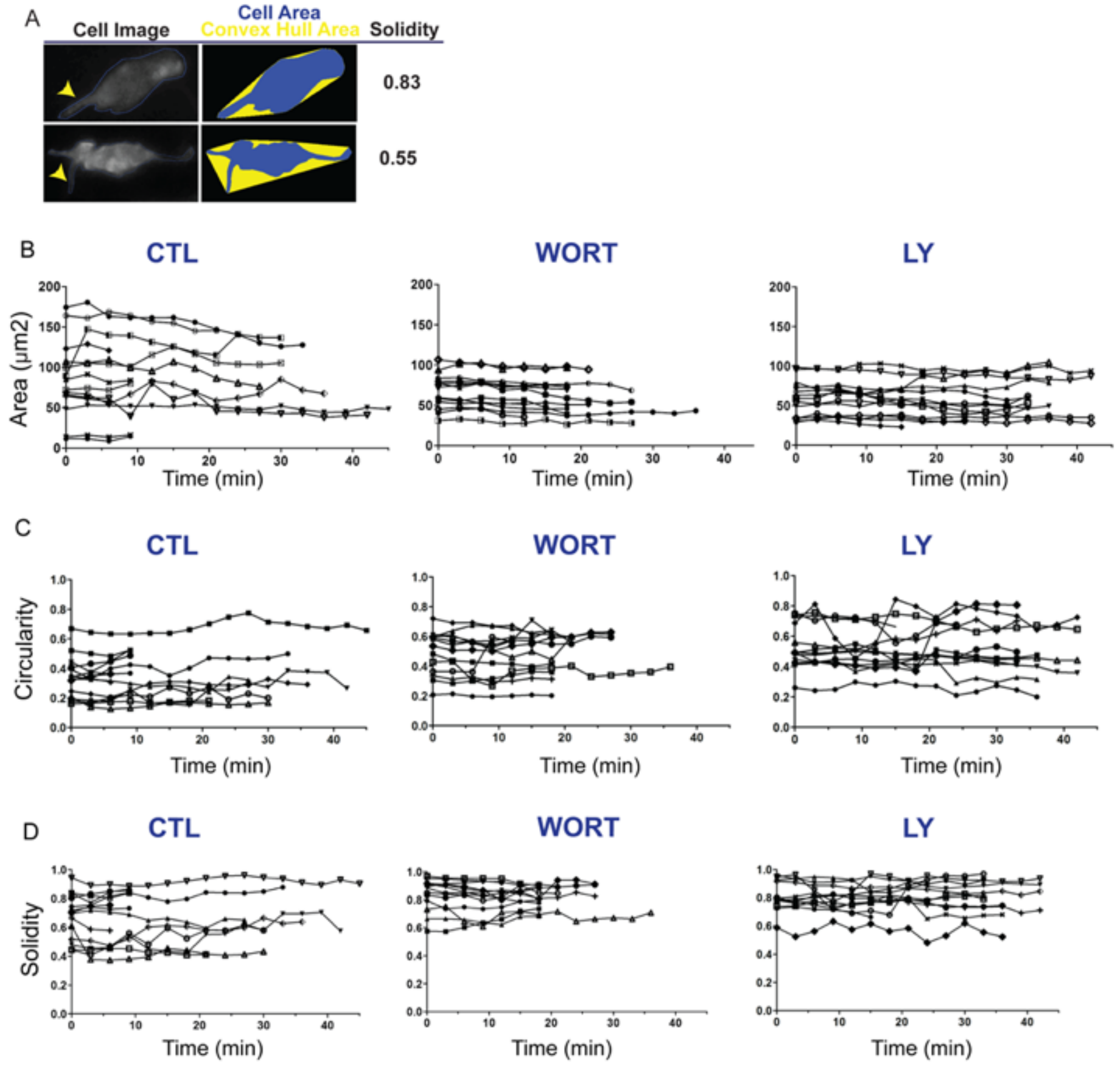

Figure S8. Single cell plots of cell morphology time series. Related to Figure 5.

(A) Schematic illustration showing the impact of cell protrusions on the morphology parameter solidity. Broad protrusions (upper right, arrowhead) increase both cell and convex hull area. Narrow protrusions (lower right, arrowhead) increase convex hull area while minimally influencing cell area, thus more significantly impacting solidity as compared to broad protrusions. For cells treated and imaged as in Fig S8A, parameters of area (B), circularity (C), and solidity (D) plotted for each cell versus time (x-axis), under control (left), WORT treated (center), and Ly294002 treated (right) conditions.

Development • Supplementary information

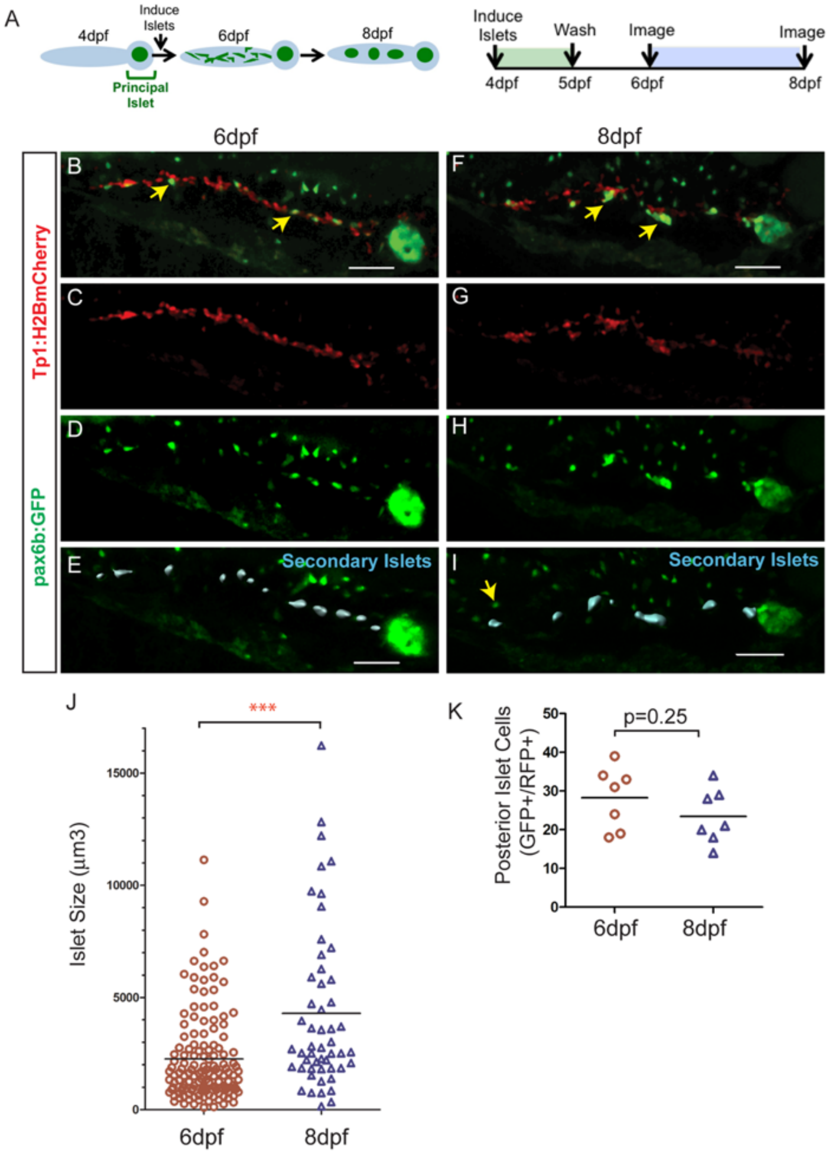

Figure S9. Quantitative assessment of islet assembly.

(A) Schematic illustration of the progression of islet assembly (left) and experimental design (right). (B-I) 3D-projections of *pax6b:GFP;Tp1:H2BmCherry* larva generated using Imaris. Dispersed mCherry$^+$/GFP$^+$-cells at 6 dpf (B, arrows, D-E) and clusters at 8 dpf (F, arrows, H,I). (E, I) Secondary islets in the posterior pancreas (blue surfaces), identified using Imaris. Nearby GFP$^+$/mCherry$^-$ cells (I, arrow) belong to the gut enteroendocrine system. Scale bar = 50µm. (J) Volumes of secondary islets, as analyzed using Imaris (blue surfaces in E, I), at 6 dpf (n=7) and 8 dpf (n=7) ($p<0.0001$, Mann-Whitney test, one-tailed). (K) GFP$^+$/ H2BmCherry$^+$ cells at 6 dpf and 8 dpf in samples as in (B) and (F) ($p=0.25$, t-test, two-tailed). The same samples were imaged at 6 dpf and 8 dpf (n=7). Results of all analyses are representative of 2 independent experiments.

Development • Supplementary information

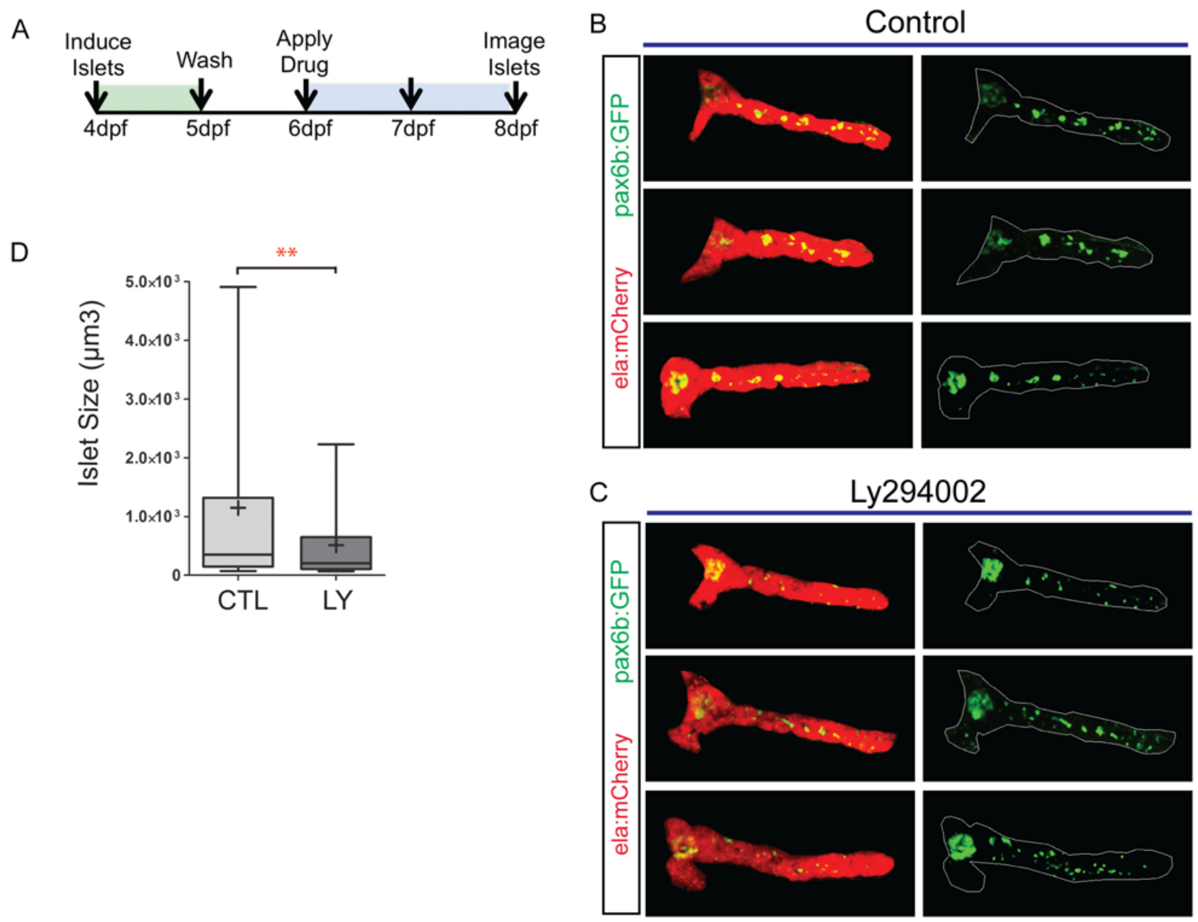

Figure S10. Image analysis to assess islet assembly. Related to Figure 6.

(A) Experiment design for pharmacological modulation of islet assembly. (B, C) Image analysis for quantitation of secondary islet size in *pax6b:GFP;ela:mCherry* larvae showing a subset of controls (B) and Ly294002-treated samples (C). Images were obtained on a Leica Sp5 and ImageJ was used for image processing (For details, see Supplementary Methods). Shown is a projection of pancreas with external signals removed (left). GFP signal alone representing endocrine pancreas (right). (D) Quantitation of secondary islet size in *pax6b:GFP;ela:mCherry* larvae following treatment from 6 dpf to 8 dpf with 15μM Ly294002 (LY), versus controls (CTL). Samples were imaged on a Leica Sp5 and analyzed with ImageJ Particle Analyser (minimum object size 50μm^3). **$p<0.01$, Mann-Whitney Test (one-tailed).(CTL, n=10 larvae, 133 objects, LY, n=8 larvae, 80 objects.)

Development • Supplementary information

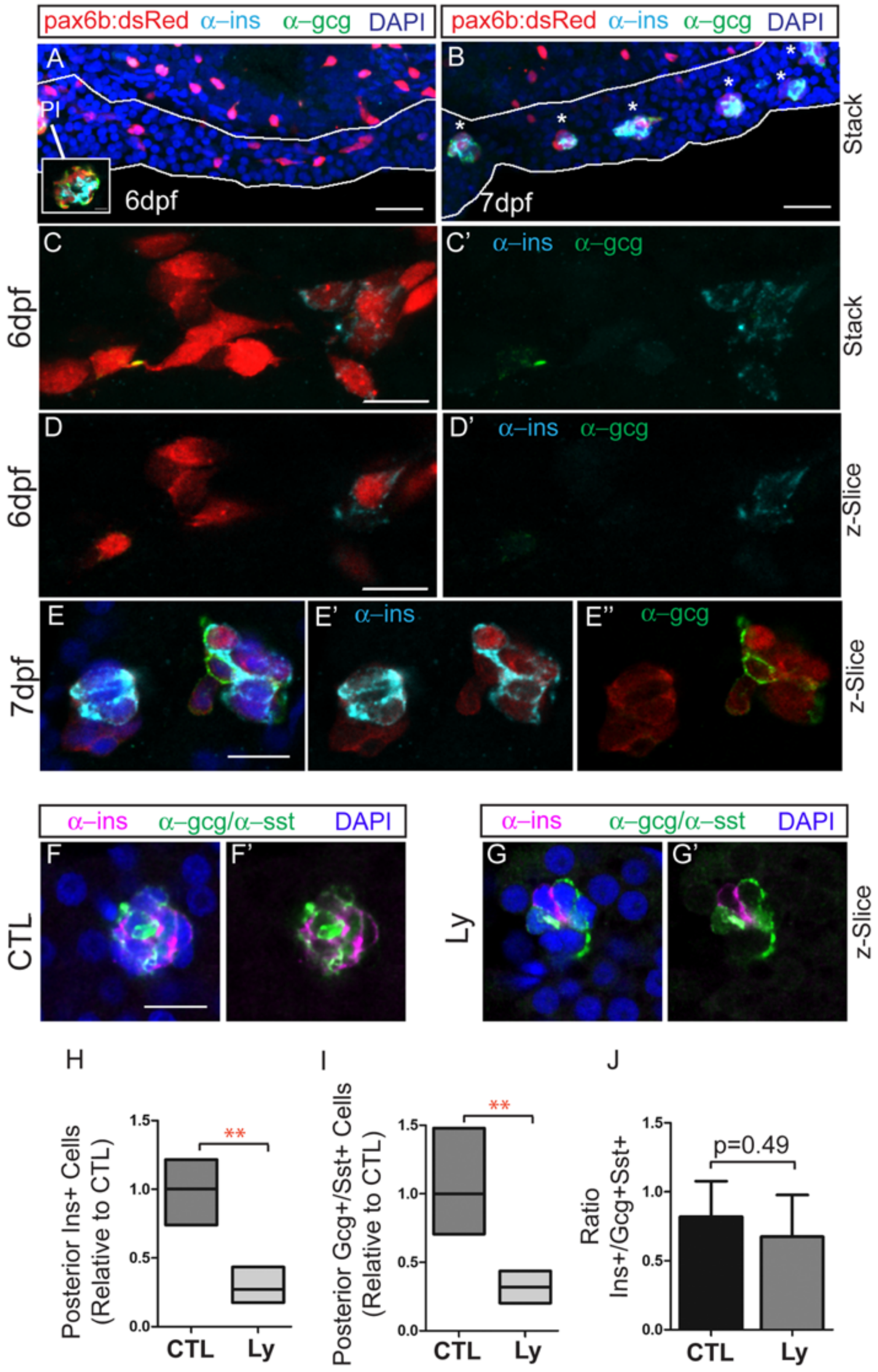

Figure S11. Expression of endocrine hormones in induced secondary islets.

Immunohistochemistry of *pax6:dsRed* larvae at 6 dpf (A, C, D) and 7 dpf (B, E) treated to induce secondary islets, labeled with anti-dsRed (red), anti-ins (cyan) and anti-gcg (green) antibodies. Pancreas is outlined in white (A,B). Inset in (A, white box), single z-plane view of principal islet (PI) shows robust staining with anti-ins and anti-gcg. (A,B) Scale bar = 25μm

Development • Supplementary information

(C-E) Scale bar = 10μm. (F,G) Endocrine hormone expression detected by antibody staining at 8 dpf, following islet induction in controls (CTL) and Ly294002-treated (LY) samples. Nuclei are counterstained with DAPI. Scale bar = 10μm. (H), (I) Relative numbers of cells from samples as in (F,G), expressing Ins (H) and Gcg (I), as compared to controls. Box plot graphs maximum to minimum, line at mean, n=4 per group, **p <0.01 (t-test). (J) Ratio of ins to gcg/sst expressing cells, plotted is the mean ± S.D.

Development • Supplementary information

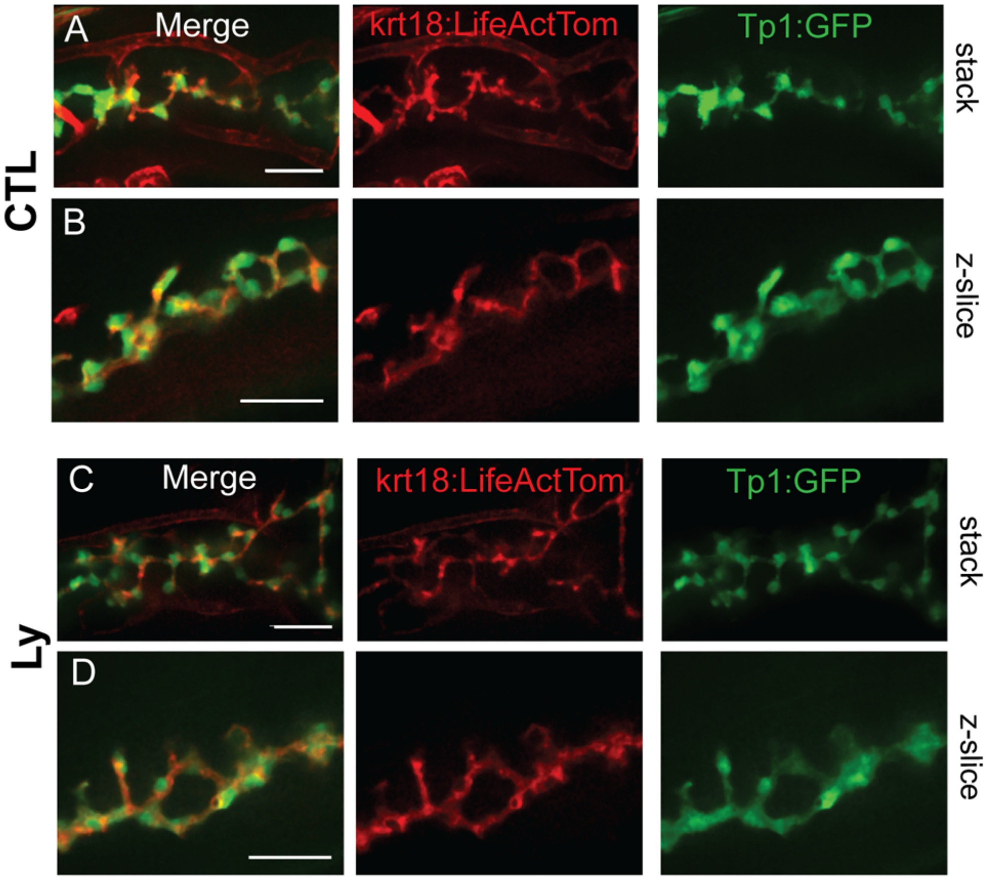

Figure S12. PI3K inhibition does not alter duct morphology.

Live-imaging of *krt18:LiveActTom;Tp1:GFP* transgenics at 7dpf, in controls (CTL, A,B) and following treatment with 15μM Ly294002 from 6 dpf to 7 dpf (Ly, C,D). (A, C) Confocal projections of image stack. (B, D) Single z-plane close-up views. Scale bar = 25μm.

Development • Supplementary information

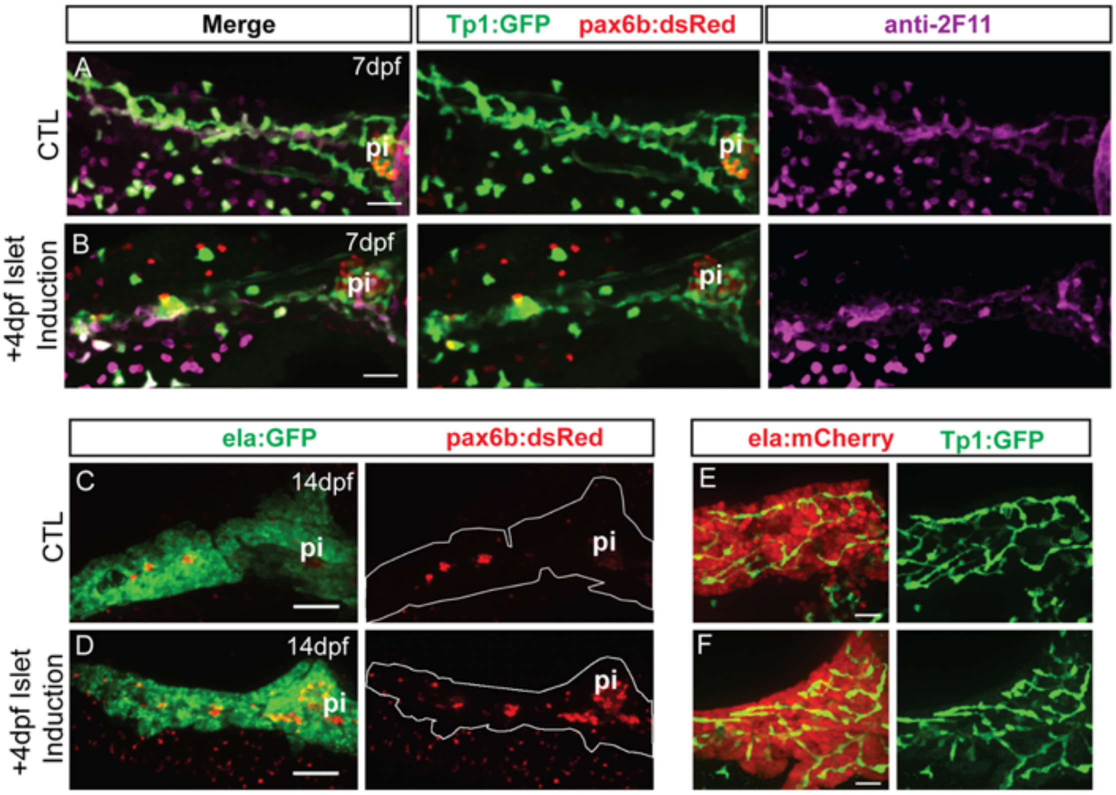

Figure S13. Islet induction transiently affects duct morphology.

(A, B) Confocal image stacks of pancreas at 7 dpf from control and islet-induced samples transgenic for *Tp1:GFP* and *pax6:dsRed*. Samples were immunostained with anti-GFP and anti-2F11. Scale bar = 25μm. (C, D) Confocal image stacks of pancreas at 14 dpf from control and islet-induced samples transgenic for *ela:GFP* and *pax6:dsRed*, immunostained with anti-dsRed. Scale bar = 100μm. (E, F) Confocal image stacks of pancreas at 14 dpf of representative control and islet-induced samples transgenic for *ela:mCherry* and *Tp1:GFP*, immunostained with anti-GFP. Scale bar = 25μm. pi, principal islet.

Development • Supplementary information

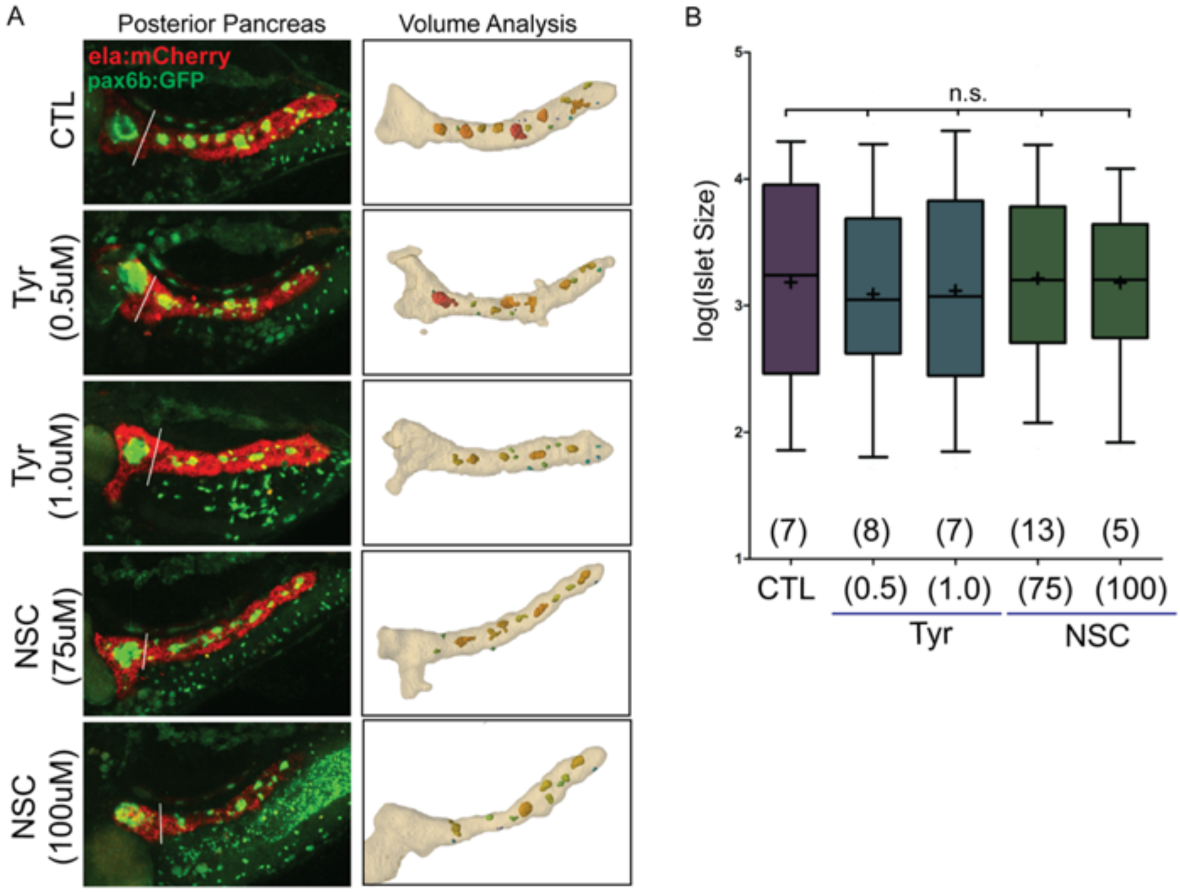

Figure S14. Impact of EGFR and Rac1 inhibition on islet assembly.

(A) Representative images from islet assembly assay and analysis (as in Fig. 6B), showing automated detection of secondary islets in control (DMSO-treated) samples and larva treated with EGFR inhibitor (Tyr) and Rac1 inhibitor (NSC) at the indicated concentrations. (B) Quantitation of secondary islets using the automated method, graphed after log transformation to display the full range of values. Boxes extends from the 25th to 75th percentile, whiskers indicate 5th and 95th percentile, line indicates the median, mean is indicated by '+'. Islet volumes in treated samples are not significantly different from DMSO-treated controls. ($p=0.6599$, ANOVA followed by Dunnett's Multiple Comparison Test). Number of larvae per group as indicated. CTL, 111 objects; Tyr (0.5μM), 115 objects; Tyr (1.0μM), 93 objects; NCS(75μM), 153 objects; NCS(100μM), 55 objects.

Development • Supplementary information

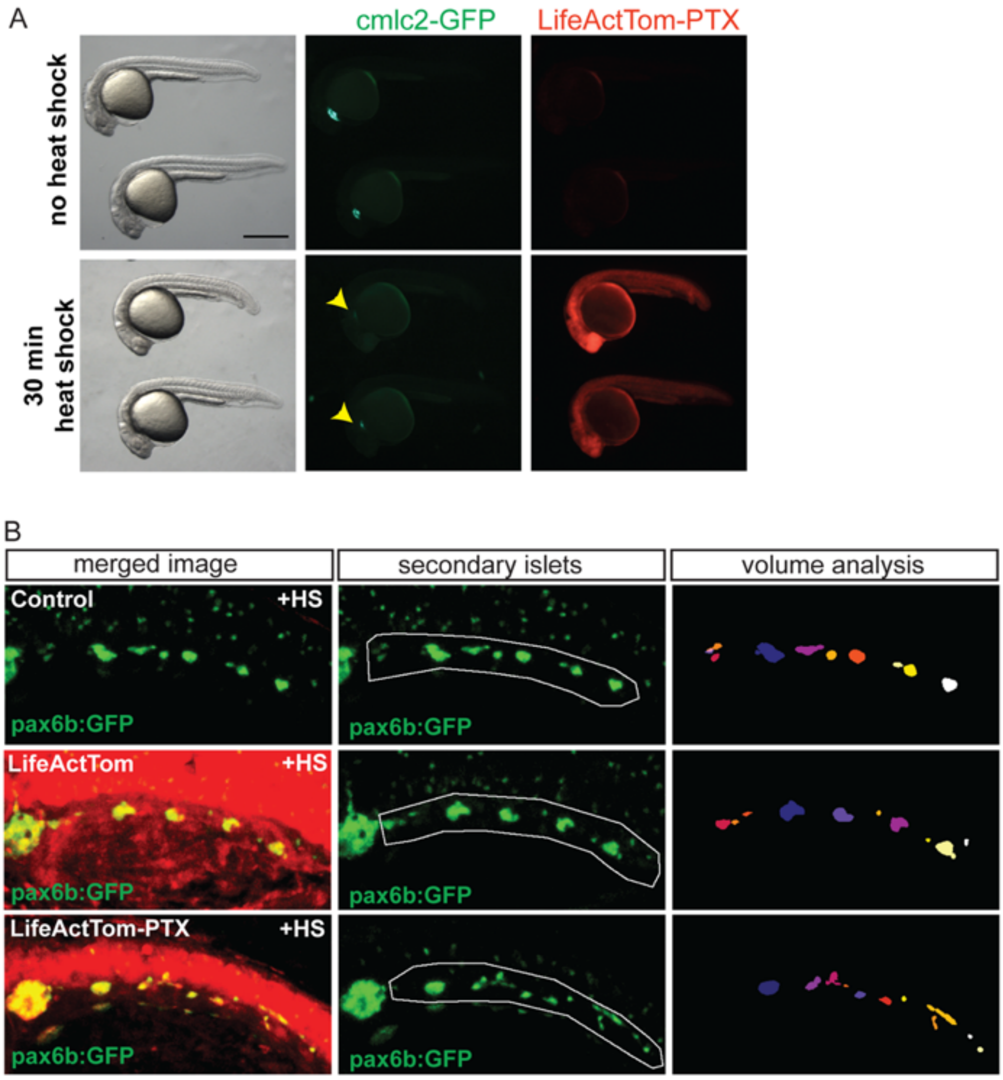

Figure S15. Heat shock induction of PTX expression impacts islet assembly. Related to Figure 7.

(A) *hsp70:LiveActTom-PTX* embryos untreated (top), or heat shocked for 30 minutes at 50% epiboly (bottom), examined at 24 hpf. Induction of PTX leads to reduced body axis length (left) and perturbed cardiac development, as evidenced by reduced *cmlc2:GFP* expression (center). LifeActTom, and by inference PTX, is ubiquitously induced following a heat shock (right). (B) Representative images of transgene expression in larvae quantitated in (7C), transgenic for *pax6b:GFP* alone (top), or *pax6b:GFP* in combination with *hsp70:LifeActTom* (middle) or *hsp70:LifeActTom-PTX* (bottom). Green channel alone (*pax6b:GFP*), posterior islets subjected to quantitative analysis are outlined (center, white line). Projection of 3D objects as identified by ImageJ plugin Particle Analyser (right).

Development • Supplementary information

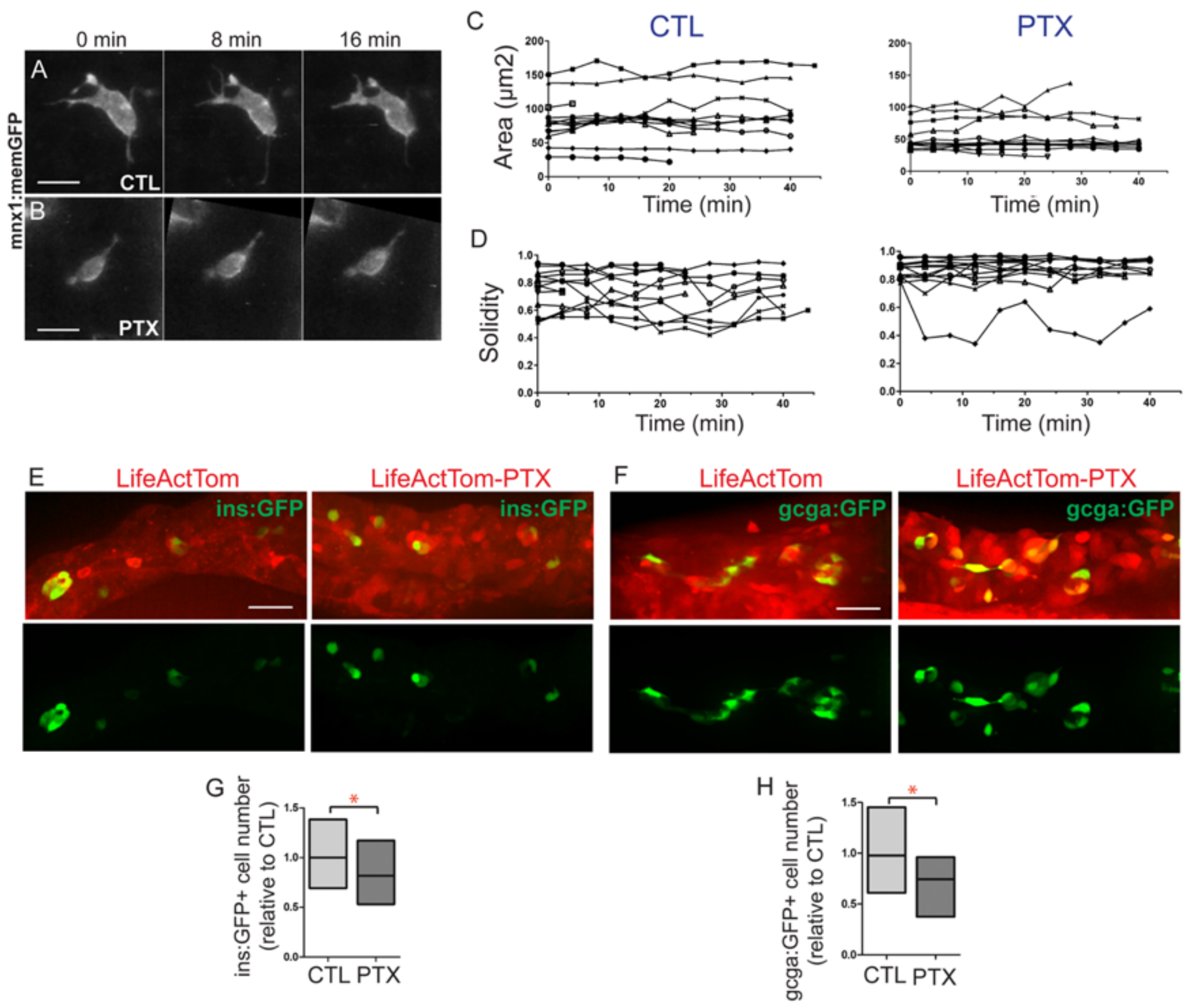

Figure S16. PTX expression impacts cell morphology. Related to Figure 7.

(A, B) Selected confocal projections, containing single cells, from time lapse series of control (A, CTL) and PTX expressing (B, PTX) transgenic larvae at 7 dpf, 4 or more hours following a heat shock. (C, D) For treated cells as in (A) and (B), morphology parameters of area (C), and solidity (D) plotted for each cell versus time (x-axis). (Samples as analyzed in Fig. 7F, G, Table S5 contains details of cells analyzed.) (E, F) Hormone-positive cells indicated by expression of *ins:GFP* (E) and *gcga:GFP* (F) transgenes in LifeActTom-expressing control, and LifeActTom-PTX induced samples, treated as in Fig. 7B. (G, H) Quantitation of cell number, expressed in relation to the controls. Box plot graphs maximum to minimum, line at mean. *$p<0.05$, t-test. (G) CTL, n=26; PTX, n=11; (H) CTL, n=12; PTX, n=12. Samples are combined from 2 independent experiments.

Development • Supplementary information

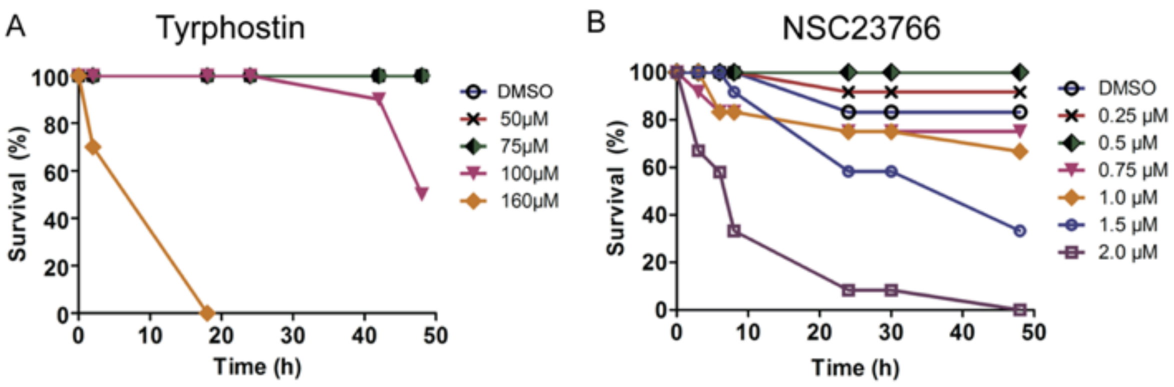

Figure S17. Toxicity curves for Tyrphostin and NSC23766.

Zebrafish larvae (5 dpf) were treated with the indicated concentrations of Tyrphostin (A) and NSC23766 (B) and observed for survival for 48h (for details see Materials and Methods and Table S7).

Supplementary Tables

Table S1. *Frequency of naturally occurring secondary islet and beta cells.*

	8dpf		2wk
# of cells	***pax6b:GFP*** (n=25)	***mnx1:memGFP*** (n=45)	***mnx1:memGFP*** (n=33)
0	9 (36%)	38 (84%)	11 (33%)
1	7 (28%)	4 (9%)	4 (12%)
2	4 (16%)	3 (7%)	7 (21%)
3	3 (12%)		3 (9%)
4	0		3 (9%)
5	2 (8%)		2 (6%)
6			2 (6%)
7			1 (3%)

(# larvae/% of total)

Samples with transgenes indicating endocrine- (*pax6b*-promoter) and early beta-cells (*mnx1*-promoter) were examined by confocal microscopy and the number of transgene-positive cells in the pancreatic tail were counted.

Development • Supplementary information

Table S2. *Quantitation of secondary islets at 2 weeks*.

Islet Frequency	
**# of islets**	**% of samples**
0-3	37%
4-6	41%
7-9	20%
10 or more	2%

Maximum Cluster Size

	None	Small	Medium	Large
**n**	2	30	36	27
**%**	2.2%	32.3%	37.8%	28.2%

Samples transgenic for *pax6b:GFP* (n=53) or *pax6b:dsRed* (n=42) were examined by confocal microscopy at 2 weeks (14-15dpf). Secondary islets in the pancreatic tail were counted and categorized as small (1-3 cells), medium (4-6 cells) or large (>7 cells).

Table S3. *Cell morphologies and dynamics in naturally occurring secondary islet cells.*

Type	Description	Frequency (%)
static	Cells in cluster with protrusions	6 (14%)
static	Isolated cells with protrusions	13 (31%)
static	Cell-Cell connection	9 (21%)
dynamic	Protrusion changes over time	12 (29%)
dynamic	Cell position changes over time	5 (12%)

Samples transgenic for *pax6b:dsRed* or *pax6b:GFP* were imaged by confocal microscopy at 13-15 dpf (as shown in Fig. 1H, S1). Configurations selected for imaging contained single isolated cells in proximity to each other or close to larger clusters (see Materials and Methods). Of 106 samples examined, 42 were imaged and followed as possible. "Static" refers to observations seen at a single time point, dynamic features occurred over >1 time point. Samples may be entered into >1 category.

Development • Supplementary information

Table S4. *Cells imaged and analyzed in control and treatment groups (Related to Figs. 5, S7, S8).*

CTL		WORTMANNIN		LY294002	
Cell #	# Frames	Cell #	# Frames	Cell #	# Frames
**1**	3	**1**	7	**1**	13
**2**	4	**2**	7	**2**	13
**3**	4	**3**	7	**3**	13
**4**	3	**4**	7	**4**	15
**5**	12	**5**	8	**5**	6
**6**	16	**6**	8	**6**	15
**7**	10	**7**	7	**7**	9
**8**	15	**8**	13	**8**	15
**9**	13	**9**	5	**9**	15
**10**	4	**10**	10	**10**	12
**11**	4	**11**	10	**11**	12
**12**	11	**12**	10	**12**	12
**13**	8	**13**	10	**13**	12
**14**	11	**14**	7		

Table S5. *Cells imaged and analyzed for control and PTX-induced groups (Related to Figs. 7, S16).*

CTL		PTX	
Cell #	# Frames	Cell #	# Frames
**1**	12	**1**	6
**2**	11	**2**	8
**3**	11	**3**	10
**4**	11	**4**	11
**5**	11	**5**	11
**6**	11	**6**	11
**7**	11	**7**	11
**8**	2	**8**	4
**9**	2	**9**	7
**10**	7	**10**	11
**11**	7	**11**	10
**12**	6	**12**	11
**13**	12	**13**	11

Table S6. *Transgenic lines in this study.*

Transgenic Line	Abbreviation	Cell type/location	Reference
Tg(ela3l:EGFP)gz2	ela:GFP	Exocrine, cytoplasm	(Wan et al., 2006)
Tg(ins:Eco.NfsB-EGFP)ml11	ins:GFP	Beta cells, cytoplasm	(Pisharath et al., 2007)
Tg(Tp1bglob:EGFP)um14	Tp1:GFP	Notch-responsive cells, cytoplasm	(Parsons et al., 2009)
Tg(Tp1:H2B-mCherry)S939	Tp1:H2BmCherry	Notch-responsive cells, nuclear	(Ninov et al., 2012)
TgBAC(NeuroD:EGFP)nl1	neurod:EGFP	Early endocrine, cytoplasm	(Obholzer et al., 2008)
Tg(P0-pax6b:DsRed)ulg302	pax6b:DsRed	Endocrine, cytoplasm	(Delporte et al., 2008)
Tg(P0-pax6b:GFP)ulg515	pax6b:GFP	Endocrine, cytoplasm	(Delporte et al., 2008)
Tg(gcga:GFP)ia1	gcga:GFP	Alpha cells, cytoplasm	(Zecchin et al., 2007)
Tg(mnx1:memGFP)ml4	mnx1:memGFP	Early beta cells, membrane	(Flanagan-Steet et al., 2005)
Tg(ptf1a:eGFP)jh1	ptf1a:GFP	Exocrine, cytoplasm	(Pisharath et al., 2007)
ela3l:mCherry	ela:mCherry	Exocrine, cytoplasm	this work
ins:mKO2		Beta cells, nuclear	this work
krt18:LifeActTom		epithelia/duct, actin filaments	this work
hsp70:LifeActTom		heat shock inducible, actin filaments	this work
hsp70:LifeActTom-PTX		heat shock inducible, PTX + actin filaments	this work

Development • Supplementary information

Table S7. *Toxicity assays for compounds used in this study.*

Compound	Concentration	Survival	Changes in morphology and/or physiology
DMSO	(control)	80-100% survived	no change
Wortmannin	2 nM	all survived	no change
Wortmannin	5 nM	all survived	no change
Wortmannin	**10 nM**	80-100% survived	reduced swimming, abdominal edema
Wortmannin	15nM	all dead	
Wortmannin	20 nM	all dead	
Ly294002	10 µM	67-100% survived	no change
Ly294002	**15 µM**	70-100% survived	no change
Ly294002	20 µM	all dead	
Ly294002	50 µM	all dead	
NSC23766	50 µM	all survived	no change
NSC23766	**75 µM**	all survived	no change
NSC23766	**100 µM**	50% survived	curved body, decreased movement
NSC23766	160 µM	0% survived	
Tyrphostin	0.25 µM	92% survived	no change
Tyrphostin	**0.5 µM**	100% survived	no change
Tyrphostin	0.75 µM	75% survived	no change
Tyrphostin	**1.0 µM**	67% survived	no change, reduced swimming
Tyrphostin	1.5 µM	33% survived	abdominal and pericardial edema, no swimming
Tyrphostin	2.0 µM	all dead	

Concentrations used are shown in **bold**. For details refer to Materials and Methods.

Table S8. *Parameters of time-lapse imaging experiments.*

Experiment	z-interval	z-range	t-interval
Fig. 3	0.8µm	64.8µm	18 min
Fig. 4H	1.5µm	32µm	19 sec
Fig. 5C (top)	1.5µm	41-54µm	4 min
Fig. 5C (bottom)	1.5µm	45µm	4 min
Fig. 7D	1.5µm	71µm	4 min
Fig. 7E	1.5µm	58.5µm	4 min
Fig. S4	0.8µm	54.4µm	9 min
Fig. S6C	0.4µm	34.2µm	3 min
Fig. S6D	0.4µm	36.9µm	20 sec
Fig. S6F	0.4µm	63µm	3 min
Fig. S6G	0.4µm	36.9µm	3 min
Fig. S6H	1.4um	68µm	3 min

Development • Supplementary information

Supplementary Movies

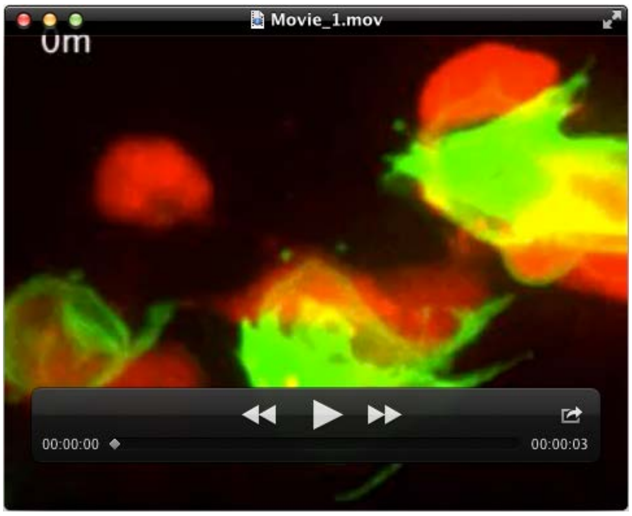

Movie 1. Time lapse of induced islet cells in *mnx1:memGFP;pax6b:dsRed* transgenic larvae at 7 dpf. (Corresponds to Fig. S4)

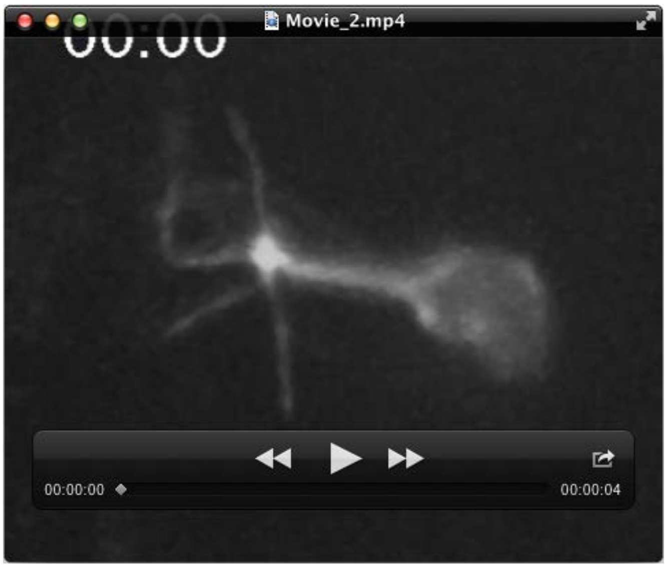

Movie 2. Time lapse of mnx1:memGFP-expressing early beta cell, imaged by spinning disc confocal microscopy at 6 dpf. (Corresponds to Fig. 4H)

Development • Supplementary information

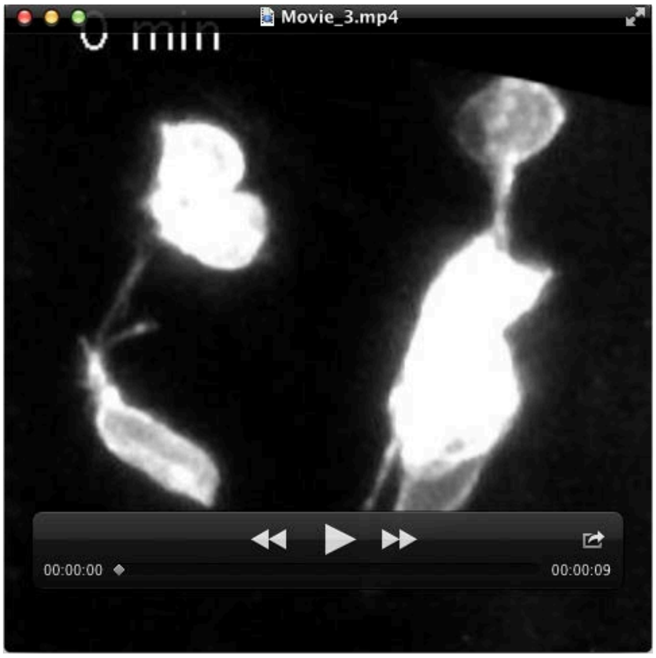

Movie 3. Time lapse of mnx1:memGFP-expressing early beta cells following induction by Notch inhibition from 4-5 dpf, imaged at 7 dpf. (Corresponds to Fig. 5C, top)

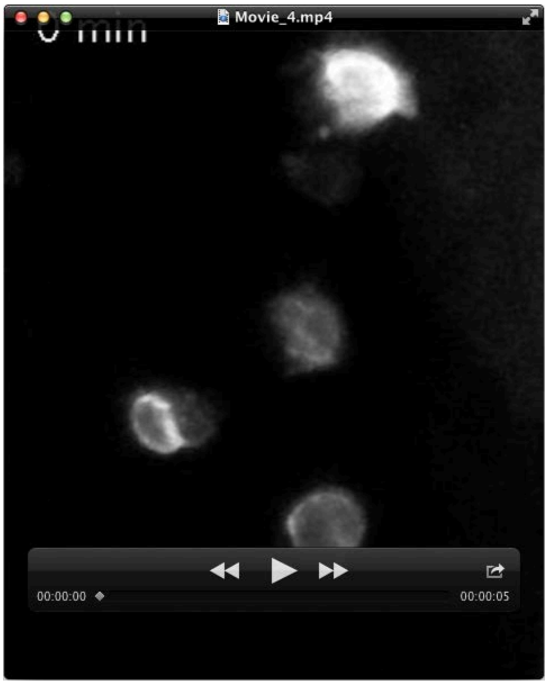

Movie 4. Time lapse of mnx1:memGFP-expressing early beta cells, following induction by Notch inhibition from 4-5 dpf, then treatment with Wortmannin for 3 hours prior to imaging at 7 dpf. (Corresponds to Fig. 5C, bottom)

Development • Supplementary information

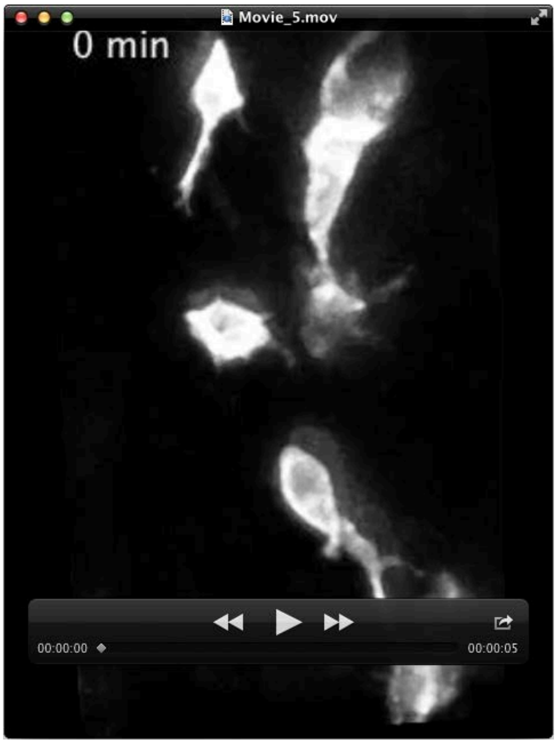

Movie 5. Time lapse series of *mnx1:memGFP;hsp:LifeActTom* transgenic larvae at 7 dpf, at 4 hours or more following a heat shock. Shown are confocal projections, GFP channel alone. (Corresponds to Fig. 7D)

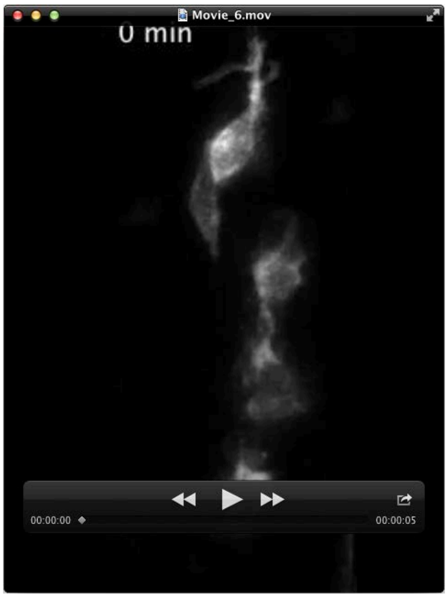

Movie 6. Time lapse series of *mnx1:memGFP;hsp:LifeActTom-PTX* transgenic larvae at 7 dpf, at 4 hours or more following a heat shock. Shown are confocal projections, GFP channel alone. (Corresponds to Fig. 7E)

Development • Supplementary information
